# High-Resolution Quantification of Focal Adhesion Spatiotemporal Dynamics in Living Cells

**DOI:** 10.1371/journal.pone.0022025

**Published:** 2011-07-14

**Authors:** Mathew E. Berginski, Eric A. Vitriol, Klaus M. Hahn, Shawn M. Gomez

**Affiliations:** 1 Department of Biomedical Engineering, University of North Carolina, Chapel Hill, North Carolina, United States of America; 2 Department of Cell and Developmental Biology, University of North Carolina, Chapel Hill, North Carolina, United States of America; 3 Department of Computer Science, University of North Carolina, Chapel Hill, North Carolina, United States of America; 4 Department of Pharmacology, University of North Carolina, Chapel Hill, North Carolina, United States of America; 5 Lineberger Comprehensive Cancer Center, University of North Carolina, Chapel Hill, North Carolina, United States of America; Kings College London, United Kingdom

## Abstract

Focal adhesions (FAs) are macromolecular complexes that provide a linkage between the cell and its external environment. In a motile cell, focal adhesions change size and position to govern cell migration, through the dynamic processes of assembly and disassembly. To better understand the dynamic regulation of focal adhesions, we have developed an analysis system for the automated detection, tracking, and data extraction of these structures in living cells. This analysis system was used to quantify the dynamics of fluorescently tagged Paxillin and FAK in NIH 3T3 fibroblasts followed via Total Internal Reflection Fluorescence Microscopy (TIRF). High content time series included the size, shape, intensity, and position of every adhesion present in a living cell. These properties were followed over time, revealing adhesion lifetime and turnover rates, and segregation of properties into distinct zones. As a proof-of-concept, we show how a single point mutation in Paxillin at the Jun-kinase phosphorylation site Serine 178 changes FA size, distribution, and rate of assembly. This study provides a detailed, quantitative picture of FA spatiotemporal dynamics as well as a set of tools and methodologies for advancing our understanding of how focal adhesions are dynamically regulated in living cells. A full, open-source software implementation of this pipeline is provided at http://gomezlab.bme.unc.edu/tools.

## Introduction

Focal adhesions (FAs) are dynamic, multi-component protein complexes that serve as points of integration for both mechanical and chemical signaling, while playing a central role in a variety of processes including cancer metastasis, atherosclerosis and wound healing [1], [2], [3]. Characterizing how these structures dynamically change is essential for understanding cell migration, which requires that adhesions are continuously remodeled as the cell moves forward. During motility, new adhesions are born at the leading edge of a protruding lamellipodia. They then enlarge and are either disassembled at the base of the protrusion in a process known as adhesion turnover, or become longer-lived structures that are eventually dismantled in the retracting tail at the rear of the cell [4], [5], [6]. In this cycle as well as other FA-mediated processes, FA dynamics are highly regulated by structural and signaling molecules [7], [8], [9]. Alterations in the balance of these regulating factors plays a key role in adhesion turnover and thus in adhesion signaling and normal cell function.

Microscope imaging of FAs underlies a significant portion of our current understanding of adhesion dynamics, with methods such as total internal reflection fluorescence microscopy (TIRF) providing high-resolution images suitable for quantitative analysis[10]. However, challenges in image capture and downstream analysis have generally led to the characterization of only a relatively small number of hand-picked adhesions within any given cell [7], [8], [11], [12], [13]. Recent technical and methodological improvements have allowed for the automated detection and characterization of focal adhesions for high-throughput screening studies. For instance, Paran and colleagues [14] have reported on the use of a high-throughput high-resolution imaging system to screen a plant extract library for effects on adhesion morphology and distribution. The same high-throughput imaging system was used to perform multicolor analysis on various adhesion components [15] and this system was used in an siRNA screen against adhesion related genes [16]. In these studies, researchers were able to obtain molecular signatures of protein components within focal adhesions, resolve sub-domains within adhesions, and identify clusters of genes that had similar effects on focal adhesion morphology and placement. These studies demonstrate the power of identifying and characterizing large numbers of adhesions within a cell. However, as the approaches used in these studies relied on cell fixation, critical aspects of focal adhesion biology, including their spatiotemporal dynamics, were lost.

Here, we describe a novel system for the quantification of focal adhesion dynamics. This approach utilizes high-resolution (60x oil-immersion) time-series images of living cells generated with TIRF. Image sequences are processed through an analysis system that identifies individual adhesions based on user-defined criteria, tracks their movement through time and collects associated properties concerning their location, shape, size and intensity. As adhesion properties throughout the lifetime of each adhesion are quantified in this approach, a thorough picture of global adhesion spatiotemporal behavior is captured.

To demonstrate the capabilities of this computational approach, we focus on characterizing adhesions via the molecular scaffold protein Paxillin, a core constituent of focal adhesions commonly used in adhesion imaging [17]. Specifically, in this study we use our image analysis system to characterize FAs labeled with EGFP-Paxillin, generating high-resolution data sets of adhesion distribution, morphology, and turnover in migrating NIH 3T3 fibroblasts. The results demonstrate that we can analyze adhesions in an unbiased manner, with 10^3^–10^4^ adhesions analyzed per cell. With wild-type Paxillin as a baseline for comparison, we use our system to detect alterations in adhesion spatiotemporal properties in response to the S178A mutation on Paxillin. Through this analysis we show that the loss of this single phosphorylation site affects adhesion site formation, size and assembly rates. We also verify the broad applicability of the analysis system by also applying the methods to examine time-lapse movies of EGFP-FAK. We are also making the analysis system available under an open source license, to allow the community to use our methods to analyze new experimental systems. These results illustrate the benefit of automated large-scale characterization of adhesion properties and behaviors, allowing both large and subtle differences to be readily detected.

## Results

### Quantitative Analysis of Focal Adhesion TIRF Images

**Figure 1:**
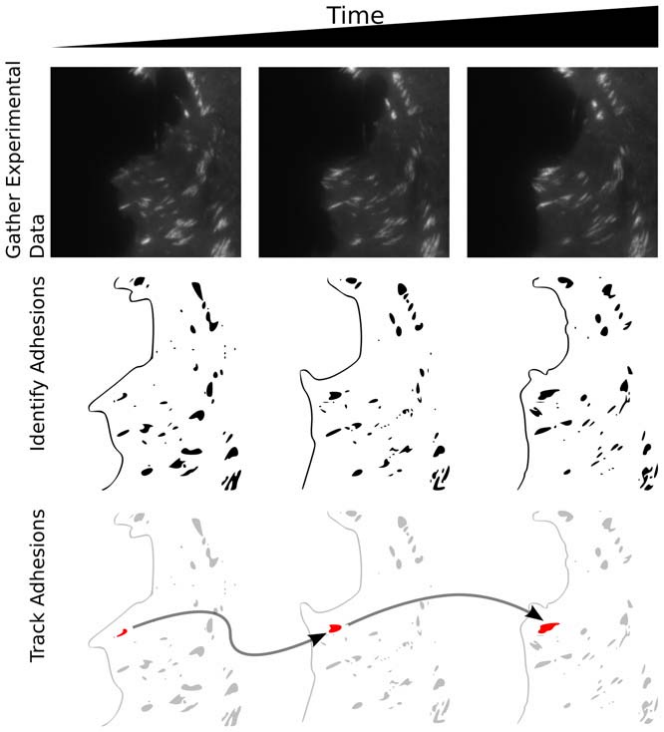
Automating the analysis of focal adhesion images requires a multi-stage pipeline. The first row shows several representative images of fluorescently labeled Paxillin using TIRF microscopy. In the second row, a cartoon depiction of the segmented adhesions and the cell edge are shown. Identification of the adhesions in each image allows a set of characteristic morphological and fluorescence intensity-based features to be extracted. The third row shows a single adhesion (highlighted in red) being tracked through the short sample time course. The properties of each adhesion are tracked over time, allowing the large scale dynamics of FA to be determined.

To quantify aspects of focal adhesion spatiotemporal dynamic behavior, we generated an NIH 3T3 fibroblast cell line expressing both EGFP-Paxillin, to label FAs, and a myristoylated-Red Fluorescent Protein (myr-RFP), to identify the cell edge. Cells were plated on fibronectin and imaged with TIRF for 1–4 hours. We then quantified FA dynamics through a multistage image analysis pipeline (Figure 1). Briefly, after high-pass filtering, FAs were identified and segmented with a watershed-based algorithm (see Methods). Characteristics of adhesions identified and quantified at each timepoint included properties such as area, position and fluorescent Paxillin intensity. Dynamic properties of adhesions, such as velocities and changes in fluorescent intensity, were also determined by tracking and measuring adhesion properties across time steps/images. At each consecutive time step the appearance of new adhesions, called birth events, and the disappearance of adhesions, called death events, were similarly identified and recorded by the software.

**Figure 2:**
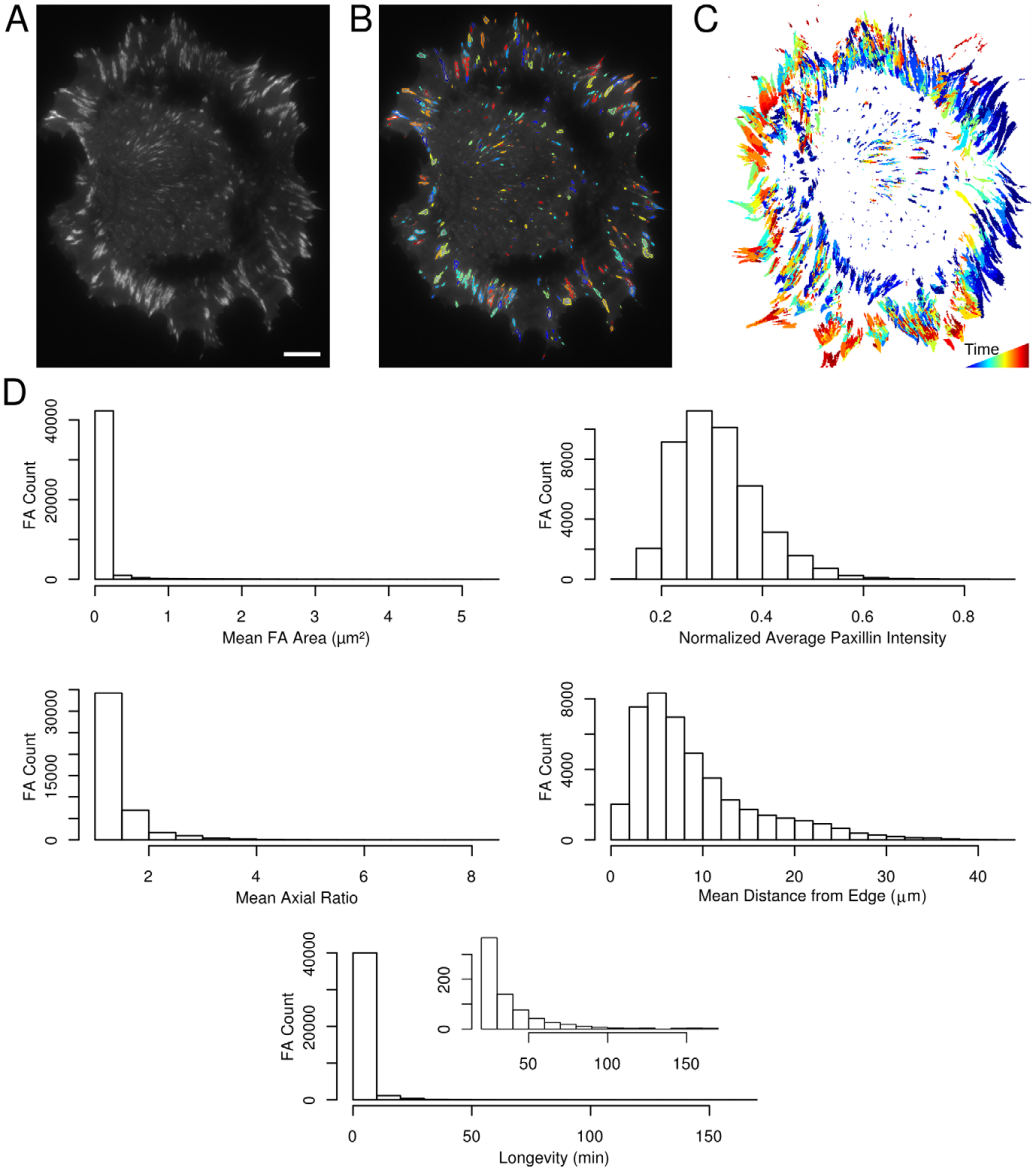
Applying quantitative image processing methods to FA images allows comprehensive characterization of FA properties. (A) One frame from a 200 minute movie of NIH 3T3 cells expressing GFP-Paxillin (the scale bar represents 10 µm). (B) The same cell as in (A), with each adhesion outlined in a different color. (C) The entire set of adhesions in an experiment can be visualized by overlaying the adhesions from each microscopy image using a different color for the set of adhesions at each time point. This example includes the adhesions from 198 images. (D) A range of properties can be extracted from the segmented FA. Five samples are provided. The area histogram was filtered to only include adhesions with areas less than 5 µm^2^. The axial ratio histogram was filtered to only include adhesions with an axial ratio of 8 or less. The longevity histogram includes all adhesions, while the inset only includes adhesions with longevity greater than 20. The histograms include data from 21 cells.

An example of the segmentation results and characteristic properties are shown in Figure 2. The segmentation methods successfully identify the adhesions in each image regardless of the background Paxillin fluorescence intensity (Figure 2A, B). The dynamic nature of the adhesions during this experiment is clear when all the adhesions identified are shown superimposed in a single image (Figure 2C). The results also show several general properties of the adhesions in wild-type cells (Figure 2D). In general, adhesions are less than 0.2 µm^2^ in size, have axial ratios less than 3 and exist for less than ten minutes, although there are many adhesions that live longer. Both Paxillin fluorescence intensity and the position of the adhesion centroids with respect to the cell edge have skewed distributions. These results demonstrate the capabilities of our system to provide high-resolution and unbiased assessment of FA behavior.

### Kinetics of FA Assembly and Disassembly

**Figure 3:**
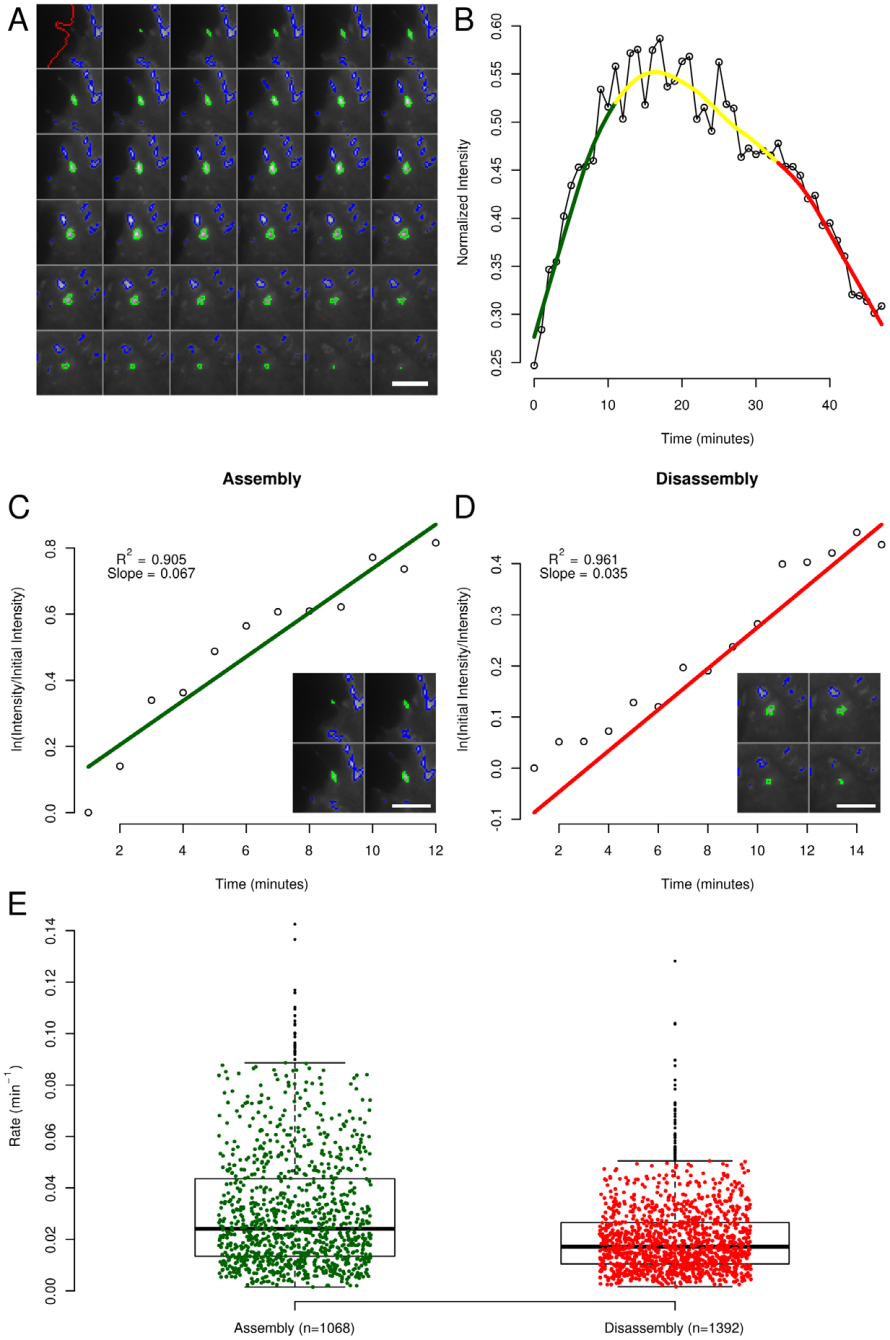
Automated measurement of focal adhesion dynamics. (A) Each of the adhesions in the cells is tracked, allowing the position and properties of single adhesions and populations to be assessed. Here a single adhesion (in green), the surrounding adhesions (in blue) and the cell edge (in red) are followed for 49 minutes. The cell edge is only outlined in the first frame. The scale bar is 10 µm. (B) The intensity of EGFP- Paxillin in the tracked adhesion in (A) through time. The green, yellow and red lines are smoothed using the Lowess algorithm and correspond to the assembly, stable and disassembly phases, respectively. (C) The normalized log-linear fit of the Paxillin intensity through time during the assembly phase of the adhesion in part (B). The inset depicts several of the images from which the Paxillin intensity was gathered. (D) The normalized log-linear fit of the Paxillin intensity through time during the disassembly phase of the adhesion in part (B). The inset depicts several of the images from which the Paxillin intensity was gathered. (E) The assembly and disassembly rates for adhesions whose Paxillin intensity curve fits have R^2^ values of 0.9 or greater. The top and bottom lines of the boxes indicate the 3rd and 1st quartiles respectively, while the bold central lines indicate the median values. The whiskers extend up to 1.5 times the interquartile range.

Of particular importance for understanding FA functions is the assessment of adhesion behavior through time. Figure 3A–D shows the methods used in determining FA assembly and disassembly rates for individual adhesions. Figure 3A depicts an image series of a single adhesion (highlighted in green) from birth, through maturation and stability, and on to death. Using time series information, we quantified the normalized intensity of each adhesion over its lifespan (Figure 3B). Readily apparent are the log-linear assembly and disassembly phases, which are automatically fit to a log-linear model (see Methods for details). Our results are consistent with previous work showing that adhesions assemble and disassemble with log-linear progression [7]. Specifically, we found that the log-linear fits for most of the adhesions produced R^2^ values above 0.7 (Figure S1). Note that the smaller number of adhesions analyzed relative to Figure 2 is due to the need for a minimum adhesion lifetime (>20 minutes) as well as other requirements needed for the accurate quantification of assembly and disassembly rates (see Methods). In the example shown in Figure 3, a log-linear approximation describes 90.5% and 96.1% of the variance in the rates of intensity increase and decrease, respectively (Figure 3C, D). In between these two phases we define a stationary/mature phase where intensity remains relatively stable (Figure 3B).

We used our system to characterize the rates of FA assembly and disassembly by repeating the analysis detailed in Figure 3A–D on all adhesions identified in the EGFP-Paxillin data set by our software (n = 21 cells). Results were focused on FAs having lifetimes of at least 20 minutes, where the detected assembly or disassembly rate is positive and the p-value of the rate model is below 0.05 (Figure 3E). The mean rate of assembly of 0.031±0.023 min^−1^ is 55% greater than that of disassembly (0.020±0.014 min^−1^). While these average rates are slower than earlier published reports, the values determined in previous studies were estimated from far fewer measurements (typically dozens of adhesions) and can be found within the variance of our data set. Thus, this automated computational approach provides a comprehensive picture of the breadth of adhesion assembly and disassembly dynamics without biasing analysis toward any particular subset of adhesions.

### Spatial Properties of FA Assembly and Disassembly

**Figure 4:**
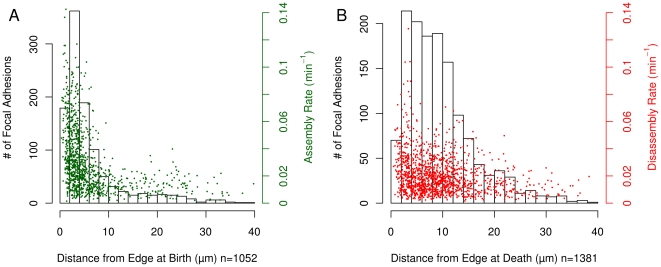
Spatial properties of FA positions at birth and death. (A) The majority of adhesions are born within 5 µm of the cell edge and the greatest variance in assembly rates are also observed in this 5 µm band. (B) The distribution of the distance of death location from the cell edge indicates that adhesion disassembly typically occurs along a broader band from the cell edge as compared to the position at adhesion birth. Also, the variance in disassembly rate is roughly the same regardless of the position at adhesion death. This data was collected from 21 EGFP-Paxillin cells.

In addition to estimation of assembly and disassembly rates, the analysis pipeline also collects spatial properties of FAs, allowing spatial aspects of FA behavior and dynamics to be similarly studied. Using the same set of experiments used to determine the kinetics of assembly and disassembly, we asked where, relative to the cell edge, adhesions tend to be born/die (Figure 4). The majority (63%) of adhesions are born less than 5 µm from the cell edge, with a mean distance from the edge at birth of 6.34 µm (Figure 4A). In contrast, adhesions tend to die further from the edge with only 27% of adhesions dying within 5 µm of the edge (Figure 4B). The mean distance from the edge at death was 9.5 µm. This suggests the existence of two distinct, but partially overlapping “zones” within which preferential birth or death of FAs occurs. When looking at both FA birth/death location and assembly/disassembly rate simultaneously, we find that higher assembly rates are observed in births that occur near the edge while no obvious effect of spatial location on the rate of disassembly is apparent (Figure 4).

### Analysis of EGFP-labeled FAK adhesions

**Figure 5:**
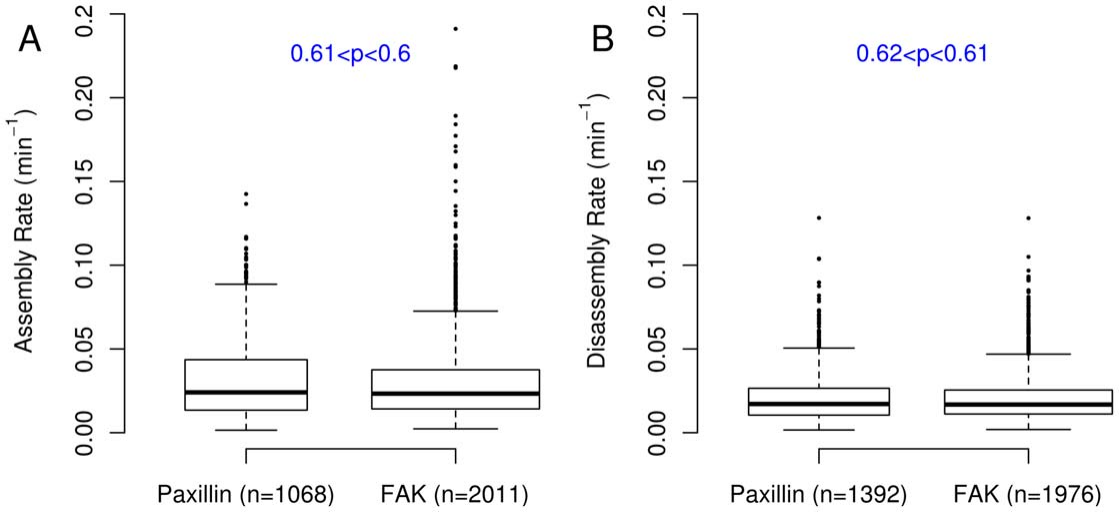
The assembly and disassembly rates of EGFP-Paxillin and EGFP-FAK adhesions are the same. The blue numbers in each plot are the p-values of the difference in median values between the EGFP-Paxillin and EGFP-FAK adhesions. P-values were calculated using the bootstrapped confidence intervals with 50000 replicates. Data from 10 cells are included.

To support the use of these methods in the study other FA proteins, we expressed FAK labeled with EGFP. After gathering time-lapse movies of 10 cells tracking the position of FAK in NIH 3T3s using TIRFM, we applied the same set of algorithms to determine the assembly and disassembly rate of the FAs. The rates of assembly and disassembly of FAs were found to be statistically indistinguishable when comparing labeled Paxillin to labeled FAK in live cells (Figure 5). In contrast, subtle but statistically significant differences in adhesion areas and axial size were found when comparing EGFP-Paxillin vs EGFP-FAK labeled adhesions (Figure S2). This result is not unexpected as different spatial and/or stoichiometric relationships are expected for both Paxillin and FAK within FAs [18], [19]. These results further indicate the capability of this system to be generally applicable to the measurement of other adhesion components besides Paxillin.

### Paxillin S178A Mutant Perturbation

The previous results establish the ability of our approach to quantify various adhesion properties and behaviors. Furthermore, the ability to identify and characterize very large numbers of adhesions provides the potential to detect changes in adhesion phenotype that are difficult or impossible to characterize manually and/or with small numbers of measurements.

As a proof-of-principle, we utilized our system to investigate the effect of a Paxillin mutation (Serine 178 to Alanine) on several aspects of FA behavior. Specifically, a principal regulatory mechanism of Paxillin is phosphorylation, with over 40 sites of phosphorylation currently identified [9]. The roles of many of these phosphorylation sites have yet to be characterized, but many of those that have been studied demonstrate strong effects on cell migration. Of particular interest is the c-Jun N-terminal kinase (JNK) phosphorylation site Serine 178 (S178). Preventing JNK phosphorylation through mutation of this Serine to Alanine, or by inhibition of JNK signaling, inhibits cell motility [20], [21]. More recently, it has been shown that phosphorylation of S178 enhances Paxillin's interaction with FAK, resulting in tyrosine phosphorylation at residues 31 and 118 [22]. Furthermore, expression of the phosphomimetic Y31D/Y118D Paxillin can rescue the S178A mutant phenotype. This and related work suggests that JNK phosphorylation of Paxillin may be an important early step in adhesion formation. However, the effects of this mutation on adhesion dynamics have not been well characterized.

**Figure 6:**
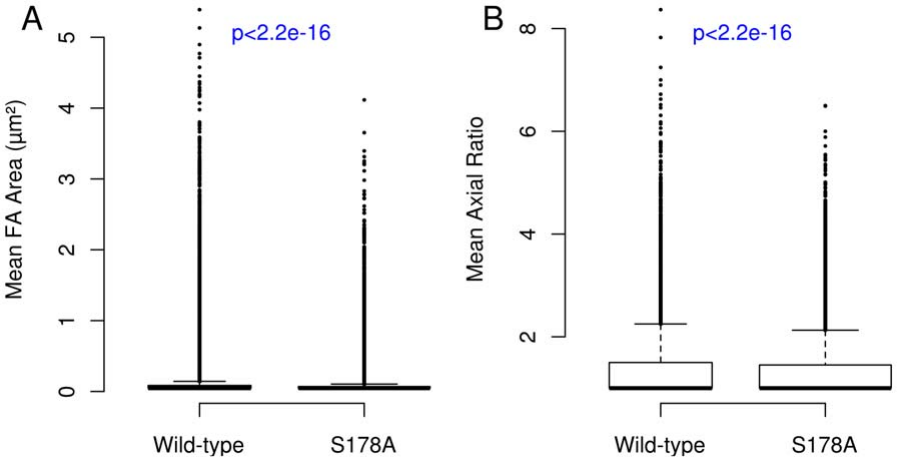
The S178A mutation in Paxillin decreases adhesion size and axial ratio. There are 44685 adhesions in the wild-type and 73305 adhesions in the S178A data sets. The p-values were calculated using the Wilcox Rank Sum test. Data from 9 cells are included in the S178A data set.

**Figure 7:**
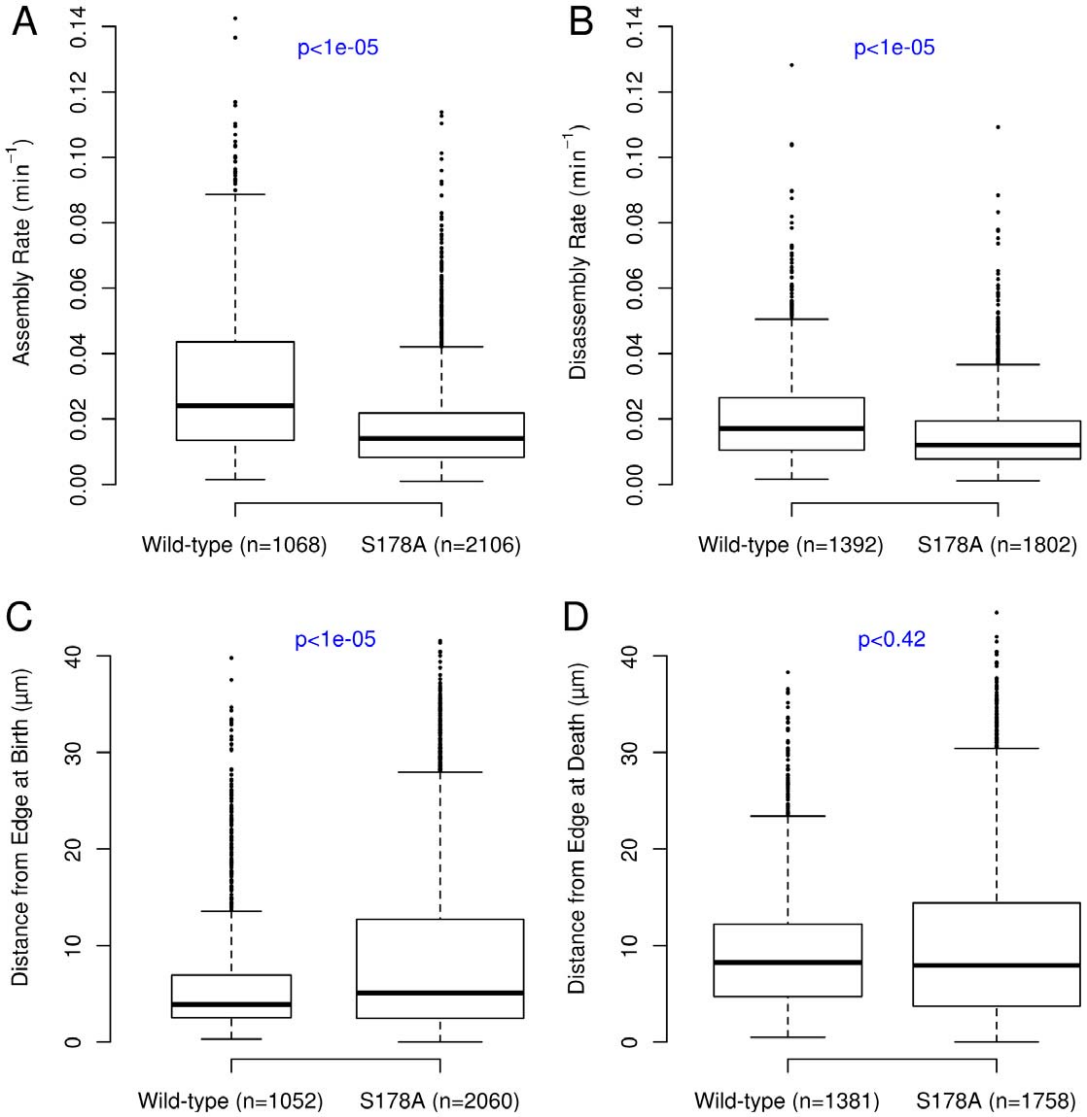
The S178A mutation in Paxillin alters adhesion assembly and disassembly. (A and B) The rate of adhesion assembly and disassembly are significantly decreased by the S178A mutation. The S178A median FA assembly rate is decreased by 42% compared to the wild-type cells, while the median disassembly rate is decreased by 36%. (C and D) The S178A mutation shifts the median adhesion birth location away from the cell edge, but has no effect on the location of cell death. The S178A median adhesion birth position is 31% greater than wild-type median birth position. The median position at adhesion death is decreased by 4% between the S178A and wild-type cells. P-values were calculated in the same manner as in Figure 5.

Using our analysis system we found that the S178A mutation induced a number of significant effects on the morphological, dynamic and spatial properties of adhesions. The mean area of the S178A mutant adhesions decreased by 23%, while the mean axial ratio decreased by 5% in the S178A mutants (Figure 6). Perhaps most relevant to the observed alterations in cell motility, there is an approximately 42% reduction in the median rate of adhesion assembly (Figure 7A). We also observe a smaller (30%) but statistically significant decrease in median disassembly rate (Figure 7B). Thus, the kinetics of FA assembly and disassembly are strongly affected by this mutation, but in a non-symmetric manner.

We previously observed that adhesions in wild-type cells have different distributions of birth and death positions relative to the cell edge. In comparison to WT cells, we find that the median distance from the edge at birth is greater by 30% in S178A mutants (Figure 7C). There is no significant difference between WT and mutant cells with regard to where an adhesion dies, suggesting that spatial aspects of the disassembly process (i.e. where disassembly occurs) is not dependent and/or sensitive to JNK phosphorylation (Figure 7D).

**Figure 8:**
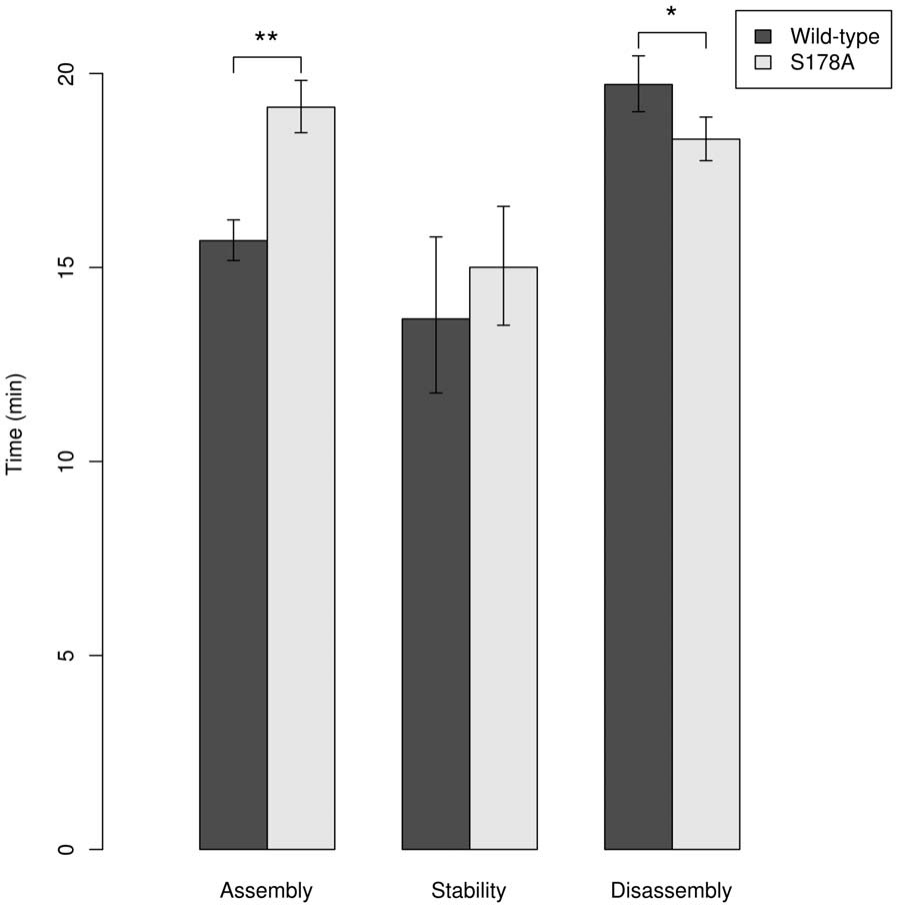
The lengths of the assembly and disassembly phases in S178A mutant FAs are significantly different from those in the wild-type, while the stability phase lengths are unaffected. The phase length values include all adhesions where the log-linear models fit with a p-value of 0.05 or less. Error bars indicate 99% confidence intervals on the mean phase length as determined through 50,000 bootstrap samples. A double asterisk (**) indicates *p*<10^−5^ and single asterisk (*) indicates *p*<0.05. Wild-type N Values: Assembly (1068), Stability (465), Disassembly (1392); S178A N Values: Assembly (2106), Stability (870), Disassembly (1802).

Finally, we compared the length of time spent in the assembly, stationary, and disassembly phases for cells expressing either WT or S178A EGFP-Paxillin. Results suggest that the S178A mutation causes adhesions to be longer-lived, spending a greater amount of time in the assembly phase than WT cells and lesser time in the disassembly phase (Figure 8). There is no difference in time spent in the stability phase. As a whole, our results demonstrate the most pronounced effects of the S178A mutation occur in the assembly phase: position at birth, assembly rate, and time spent assembling.

## Discussion

We have described the development of a set of computational tools suitable for the global characterization of FA spatiotemporal dynamics and assessing the results of network perturbation on adhesion properties and behavior. The S178A mutation was presented as a proof-of-concept perturbation study for the application of these tools to the analysis of complex FA phenotypes. Through this analysis, we were able to show that adhesion dynamics fall into distinct behavioral subtypes occurring in different regions of the cell, and that the S178A Paxillin mutant causes significant changes in FA assembly and disassembly. While requiring further investigation, these observations suggest a potential mechanism for the previously observed migration defects [20] and suggest that JNK, via Paxillin, may play a significant role in the control of the FA lifecycle.

The computational tools presented allow the entire FA life span to be analyzed. These tools include an automated adhesion detection, segmentation and tracking system; extracting a range of properties valuable for understanding FA development. All of these methods were tested using simulated data that replicated many of the observed experimental processes, confirming these methods are able to accurately quantify adhesion properties under controlled conditions (see Figure S11, Figure S12, Figure S13 and methods). The differences detected between the wild-type and S178A mutants are robust, being preserved through a range of parameter choices for the adhesion detection limit and the minimum length of the assembly and disassembly phases. The rate at which images were taken in this work (1 sample/min) also appears to be over the sampling rate needed to accurately measure the assembly and disassembly rates of long-lived adhesions (Figure S9 and Figure S10).

Our analysis system integrates methods for automatically identifying and extracting rates of FA assembly and disassembly. We find that the assembly and disassembly rates detected using these automated methods encompass the rates determined using manual methods [7], while quantifying vastly greater numbers of adhesions. We also find that adhesions labeled with an alternate adhesion marker, FAK, also allows a similar number of adhesions to be quantified and that these adhesions are similar to those detected using fluorescently labeled Paxillin. Differences in the mean rates detected by manual versus automated searches can be attributed to several factors. First, the rates determined using manual methods originate from user-specified adhesions of interest. Such adhesions may be chosen based on specific localization properties, such as selecting only those adhesions found within particular cell regions, while the presented results do not make any distinction between adhesions present in different cellular structures *a priori* (though the properties of adhesions at particular locations can be determined *a posteriori*). In addition, due to our emphasis on observing the birth, death and taking multiple samples during the assembly and disassembly phase of an individual adhesion, our rate analysis focused on long-lived adhesions, which might have different properties than those measured in studies encompassing primarily short-lived adhesions. Finally, as our software analyzes all adhesions regardless of the brightness of the adhesion, we avoid biases that may occur through, for example, preferential selection for analysis of large or highly visible adhesions. Thus, the automated methods described here greatly extend the types of adhesions that can be readily analyzed, as well as the range of properties that can be quantified.

The spatial properties of FA birth and death suggest that FAs have distinct regions where assembly and disassembly events are most concentrated. These assembly and disassembly regions overlap, but remain distinct. The greatest concentration of assembly events occurs within 5 µm of the cell edge. Previous studies in the same cell line indicate that this 5 µm range coincides with the end of the lamellipodia and the beginning of the lamella, where the structure of the actin cytoskeletal network changes significantly. Recently published data indicate that this transition, where stable actin structures differentiate into branched structures that exert force on the leading edge for protrusion, is determined by interactions between the cytoskeleton and adhesion proteins [23]. Further investigation will be required to more fully interpret this observation and its relation to the lamella-lamellipodium interface [24].

Our analysis enabled us to quantify differences in FA dynamics caused by mutation of Paxillin at a JNK phosphorylation site. Both adhesion assembly and disassembly were affected. In addition to these strong perturbations, more subtle changes in FA dynamics and localization were also detected, including a decrease in adhesion size. In agreement with our results, a recent siRNA screen of FA proteins within fixed cells that included JNK knockdown also measured decreases in adhesion size [16].

**Figure 9:**
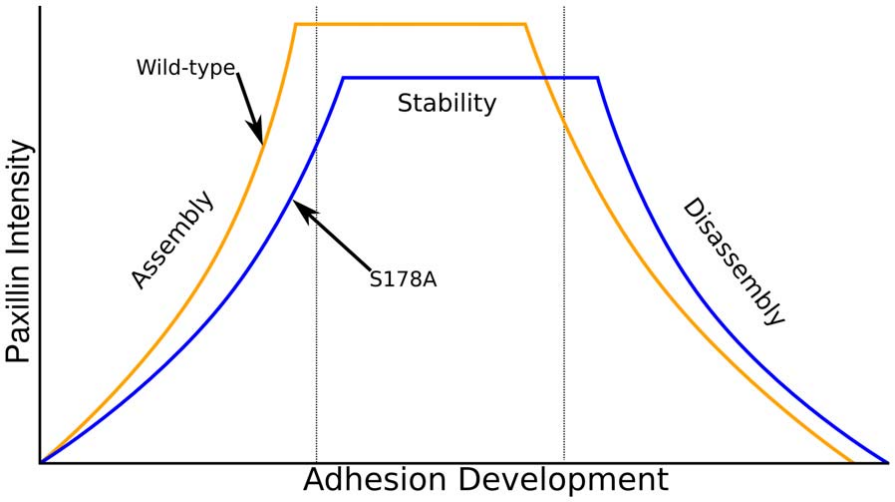
Summary of results and conceptual model of how the S178A mutant affects the adhesion life cycle. Durations and slopes are shown to scale.

Based on our results, a summary model of the FA lifecycle in both wild-type and S178A cells is depicted in Figure 9. Shown to scale, the S178A mutation shows distinct effects on both the assembly and disassembly phases of FA development, but these effects are different in magnitude. Determining what FA development signals are involved in perturbing assembly, stability and disassembly is an ongoing process, but these proof of principle TIRF experiments demonstrate the capabilities of the software analysis system to make biologically significant new observations.

Development of new and/or improved analysis modules is ongoing. In prior studies, analysis of the cell edge velocity has proven to be a robust phenotype that can be used to quantify the effect of many different perturbations to the signaling networks that control cellular motility [25]. Integration of this type of data will allow the rates of cell edge movement to be analyzed in terms of FA phenotypes. Such studies will help to bridge the gap between FA dynamics and the well-developed fields of cell edge and cytoskeletal dynamics. The data sets collected using the software also provide information about the specific properties of the adhesions during each phase of their lifecycle. There are also several types of measurements that we plan on adding to the analysis system to help quantify polarized cells behaviors, such as adhesion sliding. We also expect to continue to develop the spatial analysis methods beyond the “distance from the cell edge” measure used here. Such spatial methods will also be important in understanding polarized cell behaviors.

In summary, we have described a system for quantifying the spatiotemporal dynamics of FAs, generating highly-detailed descriptions of FA behavior based on large populations, and further enabling high-content screening methods to be applied to understanding the perturbation of FA signaling networks. The system was applied to quantifying the differences in FA development generated through a single amino acid mutation of the FA scaffolding protein Paxillin. Future studies of other FA perturbation methods with high-content analysis methods should provide a comprehensive picture of the role of FA signaling proteins in the control of FA development and localization.

## Methods

### Cell Culture

NIH 3T3 fibroblasts and 293 LinXE ecotropic packaging cells were cultured in 5% CO_2_ at 37°C in Dulbecco's modified Eagle's medium (DMEM, Mediatech) supplemented with 10% fetal calf serum, 1% L-Glutamine, and 1% penicillin-streptomycin. Fibroblasts were imaged in Ham's F-12K medium without phenol red (SAFC Biosciences) with 2% fetal bovine serum, 15 mM HEPES, 1% L-Glutamine, and 1% penicillin-streptomycin.

To make stable cell lines, retroviral vectors were transfected into 293 LinXE cells plated in 6 cm dishes with Fugene 6 (Roche) according to the manufacturer's protocol (using 18 µL of Fugene 6 and 4.5 µg of DNA). The media was replaced after 12 hours. Viral supernatant was harvested 48 hours after media replacement, passed through a .45 µm syringe filter and then added to NIH 3T3s plated at subconfluent densities at a ratio of 1∶3 (viral supernatant/normal media). Cells were infected for several rounds until they reached expression levels sufficient for live cell imaging. All of the constructs used in this study have been verified to colocalize with endogenous proteins [20], [26], [27]. No differences were detectable in the expression levels of the EGFP-Paxillin and EGFP-PaxillinS178A constructs (Figure S3).

### Microscopy

Prior to imaging, NIH 3T3s were plated onto coverslips coated with 5 µg/mL Fibronectin (Sigma) for 30 min. Fibroblasts expressing EGFP-PaxS187A required 2–3 hours to adhere to the coverslips due to a spreading defect. Immediately before being transferred to a sealed imaging chamber, complete culture media was replaced with imaging media. Imaging experiments for all cells used in this study were conducted within the first 8 hours after plating.

Imaging was performed on an Olympus IX81 motorized inverted microscope equipped with a ZDC focus drift compensator and a TIRFM illuminator, a 60X 1.45 NA PlanApoN TIRFM objective, a cooled digital 12-bit CCD camera (CoolSnap, Roper Scientific), a 100 W Mercury arc lamp, and MetaMorph imaging software. The 488 nm laser line from a Krypton-Argon ion laser (Series 43, Omnichrome) was controlled with a custom laser launch/AOTF (LSM Technologies). Imaging of the cells expressing EGFP-FAK was performed on a Nikon Eclipse Ti inverted microscope equipped with the Perfect Focus System, a TIRF illuminator, a 60X 1.45NA PlanApoN TIRF objective, a a cooled digital 16-bit EMCCD camera (QuantEM: 512SC, Photometrics), an Argon ion laser (Melles Griot) controlled with a custom laser launch/AOTF, and Nikon Elements imaging software. Images were acquired with 2×2 binning, except for images of EGFP-FAK expressing cells, which were acquired with 1×1 binning. Images were gathered once every minute. Illumination intensity was controlled with either the AOTF (TIRF excitation) or neutral density filters (epifluorescence excitation). Simultaneous TIRF images of EGFP and epifluorescence images of RFP were acquired using an 80/20 (TIRF/Epifluorescence) splitter mirror, a custom dichroic mirror (Chroma) and the following band-pass filters: EGFP (HQ 525/50); RFP (HQ580/30, HQ 630/40). In total, 21 EGFP-Paxillin, 9 EGFP-PaxillinS178A and 10 EGFP-FAK cells were included in this study. The EGFP-Paxillin experiments were conducted over four days, while the EGFP-PaxillinS178A experiments were conducted over three days and the EGFP-FAK experiments were conducted over three days.

### Image Processing

Methods to identify individual FAs were adapted from [28], with some modification. Briefly, each image taken during an experiment was high pass filtered, using a round averaging filter with a radius of 11 pixels (4.95 µm diameter). The high pass filtered images were threshholded by an empirically determined value set to identify adhesion pixels. The water segmentation method was used as described, but with the following modifications. When a pixel acts as bridge between two large adhesions, where large is defined as 40 or more pixels (1.85 µm^2^), the bridge pixel is assigned to the adhesion whose centroid is closest to the bridge pixel. Also, holes in any single adhesion were filled using the same water segmentation algorithm. Between 200 and 600 adhesions were found in each image from the experimental data. The average signal-to-noise ratio was 6.04 as calculated by dividing the mean of the adhesion intensity by the standard deviation of the backgound pixels [29]. After each focal adhesion has been identified, characteristic adhesion properties, such as those in Figure 2, are then collected.

Cell edges were found by analyzing the myr-RFP images using a method similar to that described in a prior publication [30]. This method automatically identifies a single threshold which splits the myr-RFP images into cell body and background regions. Briefly, a histogram of all the intensity values for a single image was collected and split into 1000 equal sized bins. The counts of each bin were then smoothed with the loess algorithm (Polynomial order 2, 5% of data included in each fit). This smoothed histogram has two peaks corresponding to the background region and the cell body. The local minima and maxima in the smoothed histogram are found and the two maxima at the lowest pixel intensity bins identified. The threshold for image segmentation is set to the minima between the set of maxima found in the prior step. After thresholding the image, the connected regions are identified and the regions less than 10 pixels in area are discarded. The cell edge is defined by the border pixels of the connected regions.

### FA Tracking

With the focal adhesions identified in each image of the experimental data set, another series of algorithms were designed to track the focal adhesions through each sequential image. The tracking algorithm is based on a birth-death model of a FA lifetime (Figure S4). In each sequential image a FA can either be born, continue into the next time step, merge or die. The birth-death-merge processes are detected by examining the properties extracted from the segmented adhesions. The results of this tracking algorithm are assignments of the FAs identified in each image into lineages that track the development of the FAs during the course of the experiment.

The tracking algorithm is initialized with all the adhesions detected in the first frame of the image sequence. The first step of the tracking algorithm attempts to locate FAs that correspond to one another in the next time step of the experimental data (Figure S4). This first step assumes that if a focal adhesion in the first frame overlaps with a focal adhesion in the subsequent frame, these overlapping adhesions correspond to one another. When an adhesion overlaps with more than one adhesion in the following frame, the adhesion with the greatest percentage of overlap is assigned as the match. If a FA does not overlap with any of the FAs in the following image, the FA closest to that adhesion in terms of the Euclidean distance between each adhesion's centroid is assigned as a match. Adhesions in the next frame that are not selected via either of these methods, but still overlap with an adhesion in the current frame are marked as being created via a split birth event. Adhesion births that are the result of split events are dealt with in later filtering steps. All of the living focal adhesions are assigned a corresponding FA in the following image by these percentage overlap and centroid distance rules.

This process of assigning live adhesions in one frame to corresponding adhesions in the following frame produces sets of adhesions that are predicted to merge. Some of these merge events are true merge events where one adhesion has joined with another, while others are adhesions which die, but are erroneously assigned as merge events. When a FA does not overlap with the FA it is predicted to become, this FA is assumed to have died and its lineage is ended. These adhesions are also marked as having undergone a death, which will be used in later filtering steps. For the remaining merge events where more than one adhesion has been predicted to merge in the next frame, one of the merging FA lineages is selected to continue, while the other FA lineage is predicted to end. When the adhesions predicted to merge differ in size by at least 10%, the larger adhesion's lineage is continued. If the merging FA's sizes do not differ by at least 10%, the lineage whose current centroid is closer to the adhesion centroid in the following image is predicted to continue. By this sequence of rules, each merge event is resolved so that corresponding FAs in experimental data images are determined.

After tracking live adhesions and resolving the merge and death events, the last step involves starting lineages that correspond to newly born adhesions. New lineages are started for the adhesions that had no match in the prior frame (birth events). This process of tracking the live adhesions, resolving merge and death events and starting new lineages is repeated for each image in the experimental data sequence until adhesion data from all the images have been processed. On average 2128 adhesions were tracked for each EGFP-Paxillin cells, 8145 adhesions for each EGFP-PaxillinS178A cells and 5184 adhesions for each EGFP-FAK cells. The differences in the average number of adhesions are due to longer experiments in the EGFP-PaxillinS178A and EGFP-FAK data sets. Representative videos are included in Supporting Information (Videos S1, S2, S3).

### Calculating Assembly and Disassembly Rates

With the adhesions tracked through each experiment, the characteristic properties determined for each adhesion in each frame of the time-lapse movie are collected into a set of data time series representing the properties of each adhesion through time. One type of time series follows the mean intensity of Paxillin through time, making it possible to estimate the rates of assembly and disassembly of Paxillin for each adhesion. An automated method to estimate the rates of assembly and disassembly was developed. This program automatically fits linear models to the log-transformed time series of Paxillin intensity values for both the assembly and disassembly phases of the FA life cycle.

A log-linear fitting method was adapted and extended to allow for the automated determination of assembly and disassembly phase lengths [7]. Briefly, log-linear models are fit to all the possible assembly and disassembly phases greater than or equal to a user specified minimum length. The assembly phase is assumed to occur at the beginning of the time series, whereas the disassembly phase is assumed to end with the last point in the time series. Each of the fits collected were normalized by either the first (assembly rate calculations) or last point (disassembly rate calculations) in the time series and log-transformed, as described [7].

In the second part of the algorithm, the optimum lengths of the assembly and disassembly phases were determined via a search for the maximum sum of adjusted R^2^ values of the model fits. It was assumed that the assembly and disassembly phases did not overlap. In the rare cases where there are multiple combinations of assembly and disassembly phase lengths that produce the highest sum of adjusted R^2^ values, the combination with the longest combined assembly and disassembly phase lengths is selected. The stability/maturity period was then defined as the length of time between the assembly and disassembly periods.

### Results Filtering

Several filters are used to analyze the data sets collected with these analysis methods. When determining the assembly and disassembly rates, only adhesions with at least 20 Paxillin intensity time points were analyzed. This ensured that there was sufficient data available to correctly detect the assembly and disassembly rates. Adhesions whose birth was the result of a split event with another adhesion were also excluded from the assembly rate calculations, while adhesions whose lineage ended with a merge event were excluded from the disassembly rate calculation. Assembly and disassembly fits whose linear model p-values were above 0.05, indicating that the slope of the linear model was not significantly different from zero, were also excluded from the data set.

A separate set of filters was used to determine the length of each phase (assembly, stability and disassembly) in the adhesion intensity time series data. In order to estimate the length of time an adhesion spends in the stability phase, we required that both the assembly and disassembly phases be observed. In addition, the adhesion birth could not have been the result of a split event and the death of the adhesion not the result of a merge. The filter also excluded those adhesions where the p-value of either the assembly or disassembly linear model was greater than 0.05.

### Parameter Testing

To test the sensitivity of results on parameters used for defining the threshold for adhesion detection, the minimum length of the assembly and disassembly phases and the rate of image sampling, we re-executed our analysis while varying these parameters. The threshold for adhesion detection was varied between 0.05 and 0.10, with no significant effect on the percentage change between the wild-type cells and the S178A mutant cells in either the assembly or disassembly rates (Figure S5 and Figure S6). Varying the required length for assembly and disassembly rate calculation similarly had no significant effect on percentage change between the wild-type and S178A mutant cells of the rates of assembly or disassembly (Figure S7 and Figure S8). Finally, we tested the results of changing the image sampling rate by discarding every other collected image in the same set of experiments (Figure S9). Discarding half of the images did not significantly affect the assembly or disassembly rates, but did have a slight effect on the distribution of the adjusted R^2^ values (Figure S10). From these parameter testing examples, we concluded that selection of a single set of parameters as determined by the user, provided a robust description for any of the differences between cell lines in terms of assembly and disassembly rates.

### Software Testing

In order to test the baseline performance of the algorithms, a set of gold standard images were produced with sets of FAs having specific, predefined properties. In general, validation tests consisted of simulating a time-lapse microscope field of view that mimicked the observed properties of the adhesions (Figure S11A). Since our results are consistent with prior findings based on manual methods of adhesion identification, the simulated range of properties was set to be similar to those observed in the experimental data. For all simulated experiments, a Gaussian noise model (mean 0, variance of 2*10^−3^) was used as a background to simulate the cell environment. These parameters were chosen as they produced distributions of short-lived adhesions that were empirically similar to those observed experimentally. Also, all simulated adhesions were circular and the same background noise model was used to perturb intensities assigned by the software to each simulated adhesion.

Three types of simulations were conducted: stationary, moving and kinetic. The stationary simulation consisted of simulating a field of view that included rows and columns of unmoving adhesions. The intensity of the adhesions were varied along the columns between mean intensities of 0.05 and 0.47 (95% of the detected adhesions in the experimental data fall between normalized average Paxillin intensities of 0.21 and 0.52). Ten different adhesion radii were simulated along the rows, varying between 0.5 and 5 pixels. The adhesions at low mean intensity values were not reliably discernable below intensity level 0.17. Adhesions above this level were readily detected with both the predicted intensities and sizes (Figure S11).

The moving simulation was designed to probe the tracking algorithm's performance in following adhesions of various sizes and intensities. The simulation consisted of sliding the adhesions across the field of view at different rates (Figure S12A). As expected, the smaller adhesions were more difficult to track, with a nearly linear relationship between the ability to track an adhesion moving at a certain rate and its corresponding radius (Figure S12B). As long as the adhesion is detectable, there does not appear to be any differences in the intensity versus tracking accuracy (data not shown).

To conduct the adhesion kinetics tests, sets of adhesions were simulated that went through logarithmic assembly and disassembly phases. The assembly and disassembly rates were varied by shortening or lengthening the amount of time each adhesion spent reaching its maximum intensity. The stability period in each of these adhesions was set to five frames. Assembly and disassembly lengths between 10 and 20 were all tested. In order to avoid biasing the automated assembly and disassembly phase fitting software to higher phase lengths, the minimum phase length was set to five time points during image analysis. Overall, the software was able to reliably extract both the expected assembly and disassembly rates and length of time spent in each phase (Figure S13). There were several samples in the longer phase lengths that were predicted to have substantially shorter assembly and disassembly phase lengths than that specified by the software, but these simulated adhesions were in the minority and did not significantly affect the confidence intervals around the mean assembly and disassembly lengths. These simulations further support the accuracy of results derived from applying the same sets of algorithms to the analysis of adhesions in living cells.

### Statistical Tests

Two different types of tests were used to determine the statistical significance of the differences between the adhesions in the wild-type, S178A and labeled-FAK adhesions. To compare datasets with <2000 points, bootstrap resampling was used to determine either the mean or median distribution. From these distributions the p-value was determined using the percentile method. The bootstrap method was too computationally intense to compare datasets, such as the area and axial ratio of the adhesions, with significantly more points than 2000 data points. Instead, the Wilcox Rank Sum test was used to find the p-value in these cases.

### Software Availability

The most recent version of the software system is available from the Gomez lab website (http://gomezlab.bme.unc.edu/tools). In addition to the source code, released under the BSD license, there is a sample movie that can be used to test the success of installing the analysis system. The software has been tested on Mac OS×10.5 and Ubuntu Linux 10.04.

## Supporting Information

Figure S1 **The assembly and disassembly log-linear models fit the Paxillin intensity time courses with high R^2^ values.** The red lines indicate the median length-adjusted R^2^ values.
(PNG)

Figure S2 **Adhesions labeled with EGFP-FAK are larger in mean area and have a larger axial ratio than those labeled EGFP-Paxillin.** There are 51836 adhesions in the FAK data set and 44685 adhesions in the Paxillin data set. The p-values were calculated using the same methods as Figure 6.
(PNG)

Figure S3 **There are no significant differences between the expression levels in the EGFP-Paxillin and EGFP-PaxillinS178A cell lines.** The average intensity of fluorescence inside the cell is shown in three different ways: the overall cell intensity (A), inside the cell not including the adhesions (B) and only the adhesions (C). The error bars are 95% confidence intervals determined using 50,000 bootstrap samples on the mean value.
(PNG)

Figure S4 **Flow chart for the tracking software adhesion following algorithm.**
(PNG)

Figure S5 **Changing the adhesion detection threshold does not affect the differences in the assembly rates between S178A mutant and wild-type cells.** Each boxplot contains all the adhesions with significant linear fits (linear model p-value below 0.05). The p-values in each boxplot are for the difference in medians between the wild-type and S178A data sets in each boxplot.
(PNG)

Figure S6 **Changing the adhesion detection threshold does not affect the differences in the disassembly rates between S178A mutant and wild-type cells.** Each boxplot contains all the adhesions with significant linear fits (linear model p-value below 0.05). The p-values in each boxplot are for the difference in medians between the wild-type and S178A data sets in each boxplot.
(PNG)

Figure S7 **Changing the minimum length of the assembly phase does not significantly affect the differences in the assembly rate between the wild-type and S178A mutant cells.** Each boxplot contains all the adhesions with significant linear fits (linear model p-value below 0.05). The p-values in each boxplot are for the difference in medians between the wild-type and S178A data sets in each boxplot.
(TIFF)

Figure S8 **Changing the minimum length of the disassembly phase does not significantly affect the differences in the assembly rate between the wild-type and S178A mutant cells.** Each boxplot contains all the adhesions with significant linear fits (linear model p-value below 0.05). The 95% confidence intervals on the percent change in the median assembly rate between the wild-type and S178A adhesions overlap in all minimum length settings. The p-values in each boxplot are for the difference in medians between the wild-type and S178A data sets in each boxplot.
(PNG)

Figure S9 **Reducing the time between each frame only has mild effects on the assembly and disassembly rates in the wild-type cells.** The label ‘All’ indicates that none of the images were excluded to estimate the rates, while ‘Sampled’ indicates that every other image from each experiment was discarded. To compensate for the shortened experimental time, the minimum number of points needed to determine an assembly or disassembly rate was reduced to 5 for the sampled data sets. Each boxplot describes the data from all the adhesions with significant linear fits (p-value below 0.05).
(PNG)

Figure S10 **Reducing the time between each frame only has mild effects on the assembly and disassembly rates in the S178A cells.** The label ‘All’ indicates that none of the images were excluded to estimate the rates, while ‘Sampled’ indicates that every other image from each experiment was discarded. To compensate for the shortened experimental time, the minimum number of points needed to determine an assembly or disassembly rate was reduced to 5 for the sampled data sets. Each boxplot describes the data from all the adhesions with significant linear fits (p-value below 0.05).
(PNG)

Figure S11 **Evaluation of the analysis system's ability to extract quantitative properties from simulated stationary focal adhesions.** (A) The last frame of the stationary simulation, with each adhesion outlined in a color depending on when in the movie it was born. The adhesions in blue have been detected for the longest time, while those in red and orange have been detected for the shortest amount of time. The simulated adhesions in columns 1–3 are all too faint to be reliably detected for the length of the simulation experiment, while those in column 4 are near the limit of detection. (B) The exponential distribution of adhesion longevity appears similar to that observed in the experimental data. The longevity of all the detected adhesions was correctly identified as 25 minutes. (C and D) The average adhesion intensity (C) and mean adhesion area (D) were correctly identified in the adhesions that were detected for their entire 25 minute lifespan. The red lines in C indicate the true values.
(PNG)

Figure S12 **Evaluation of the tracking algorithm's ability to follow adhesions of various sizes and speeds.** (A) A sample frame from the simulated adhesion motion experiment where the adhesions were moved at 1 pixel per frame. The top row of adhesions of only a single pixel could not be followed. (B) As the movement speed of the simulated adhesions increases, only larger adhesions can be reliably tracked.
(PNG)

Figure S13 **Evaluation of the rate and phase length detection algorithm using simulated focal adhesion images.** (A and C) The predicted median assembly (A) and disassembly (C) rates were extracted correctly by the algorithm. (B and D) The predicted lengths of both the assembly (B) and disassembly (D) were also correctly identified by the algorithm. All the red lines indicate the expected values of the properties in each plot.
(PNG)

Video S1 **Example movie showing the results of tracking the EGFP-Paxillin labeled adhesions.** The left panel shows the normalized raw experimental data, while the right hand side shows each adhesion outlined in a different color. As the movie plays, the highlighting color remains the same for each unique adhesion. The scale bar is 10 µm.
(MOV)

Video S2 **Example movie showing the results of tracking the EGFP-PaxillinS178A labeled adhesions.** The left and right panels are the same as in Video S1. The scale bar is 10 µm.
(MOV)

Video S3 **Example movie showing the results of tracking the EGFP-FAK labeled adhesions.** The left and right panels are the same as in Video S1. The scale bar is 10 µm.
(MOV)
