# Supplementary figures and images for: High-Resolution Quantification of Focal Adhesion Spatiotemporal Dynamics in Living Cells
Source: PLoS One. 2011 Jul 14;6(7):e22025. doi: 10.1371/journal.pone.0022025 (PMC3136503; doi:10.1371/journal.pone.0022025)

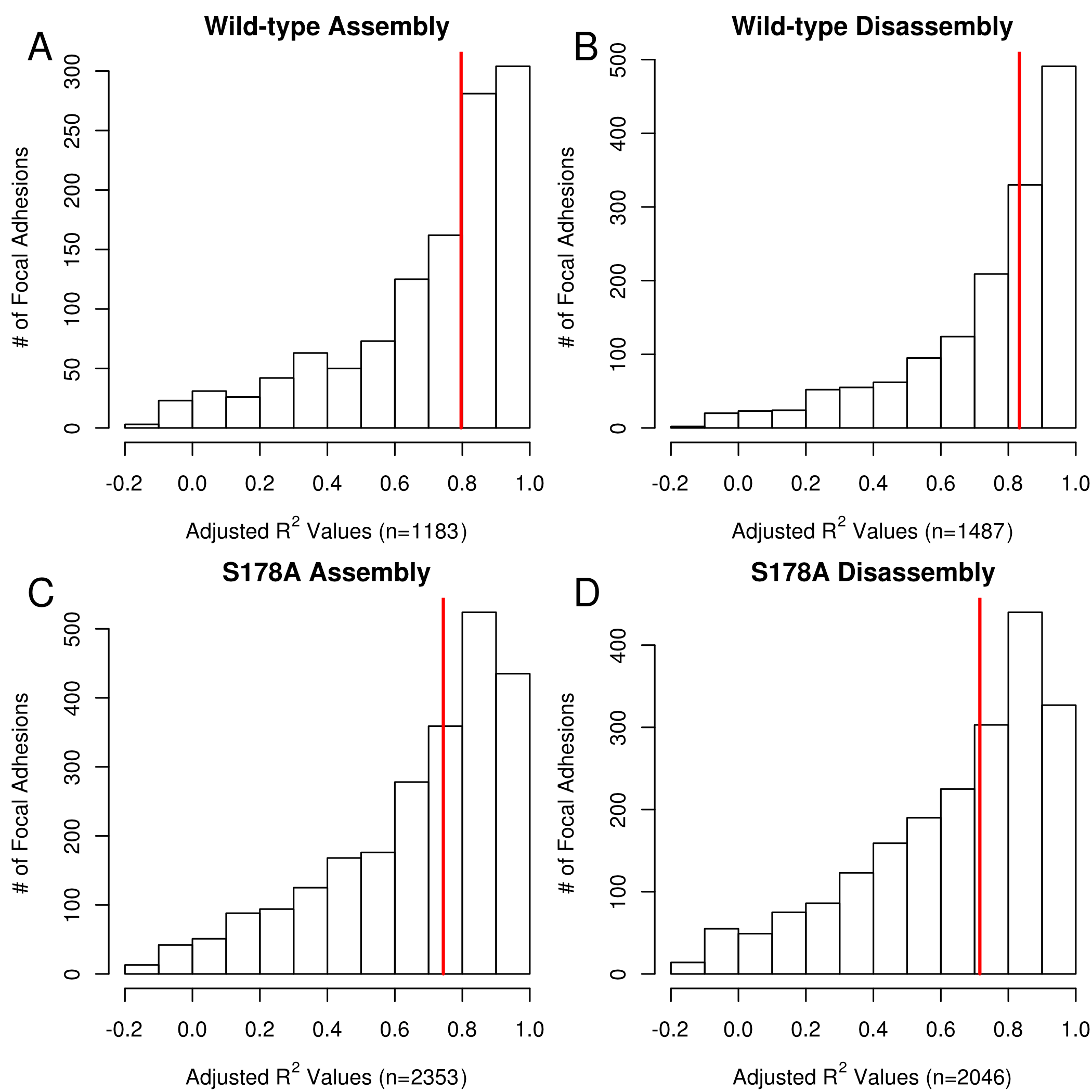

Supplement: Figure S1 — The assembly and disassembly log-linear models fit the Paxillin intensity time courses with high R2 values. The red lines indicate the median length-adjusted R2 values. (PNG) [file pone.0022025.s001.png]

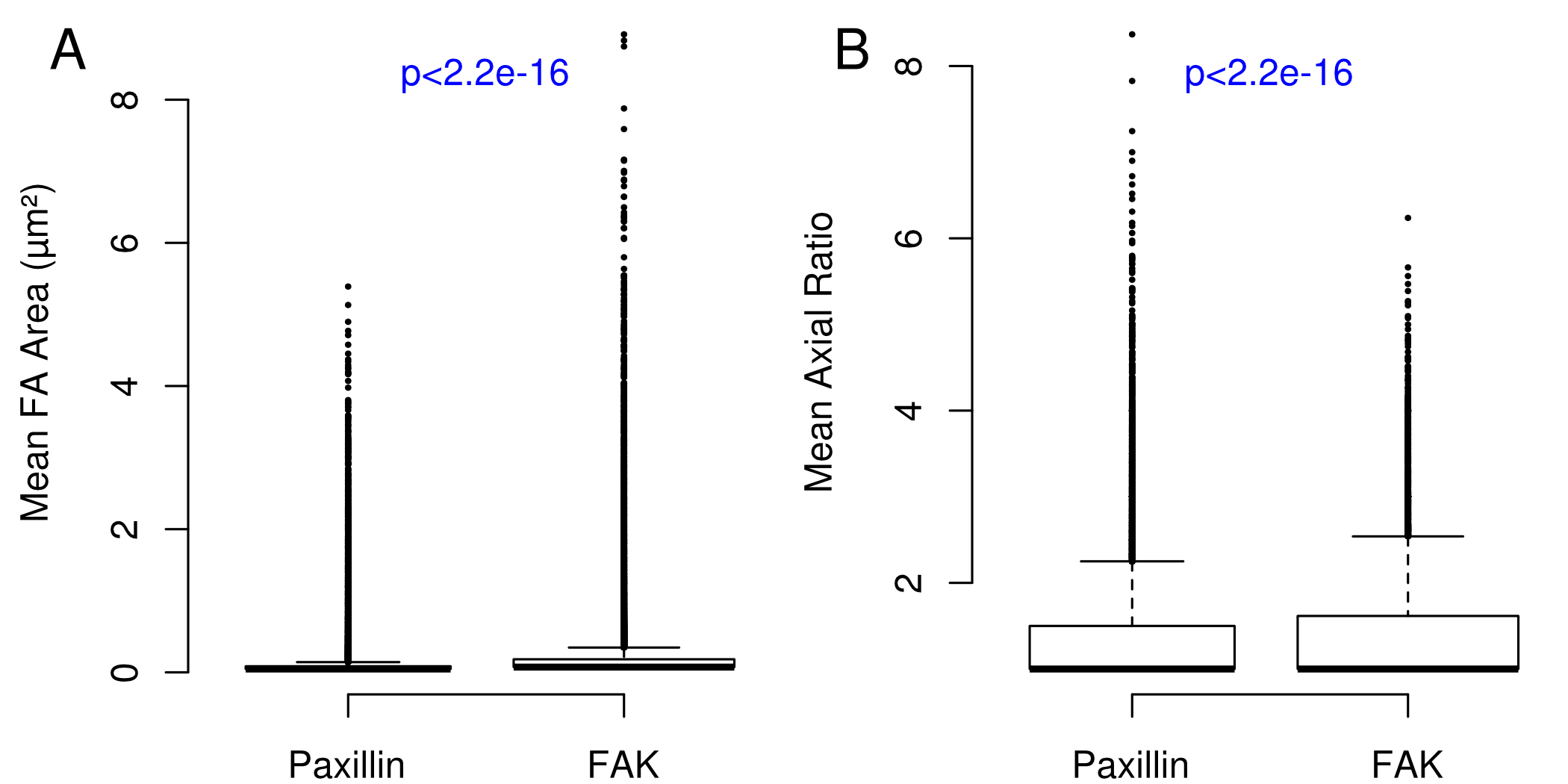

Supplement: Figure S2 — Adhesions labeled with EGFP-FAK are larger in mean area and have a larger axial ratio than those labeled EGFP-Paxillin. There are 51836 adhesions in the FAK data set and 44685 adhesions in the Paxillin data set. The p-values were calculated using the same methods as Figure 6. (PNG) [file pone.0022025.s002.png]

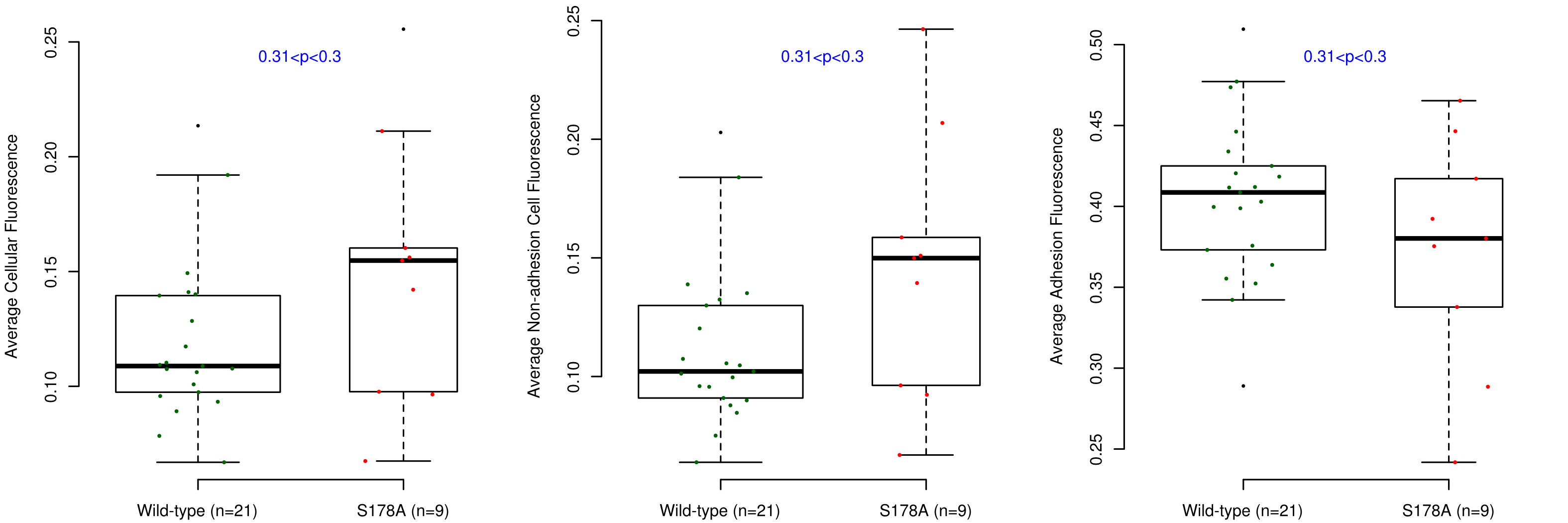

Supplement: Figure S3 — There are no significant differences between the expression levels in the EGFP-Paxillin and EGFP-PaxillinS178A cell lines. The average intensity of fluorescence inside the cell is shown in three different ways: the overall cell intensity (A), inside the cell not including the adhesions (B) and only the adhesions (C). The error bars are 95% confidence intervals determined using 50,000 bootstrap samples on the mean value. (PNG) [file pone.0022025.s003.png]

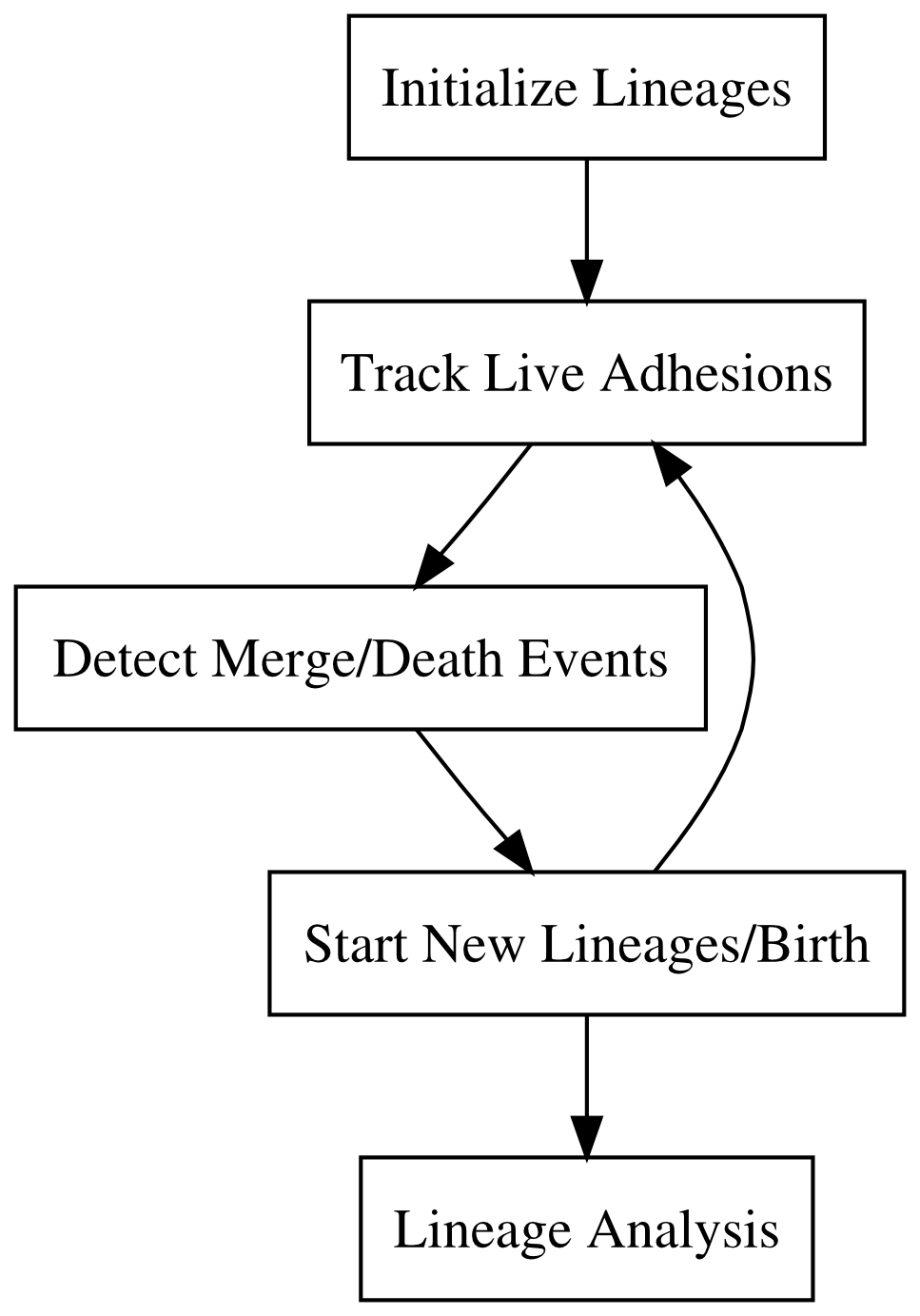

Supplement: Figure S4 — Flow chart for the tracking software adhesion following algorithm. (PNG) [file pone.0022025.s004.png]

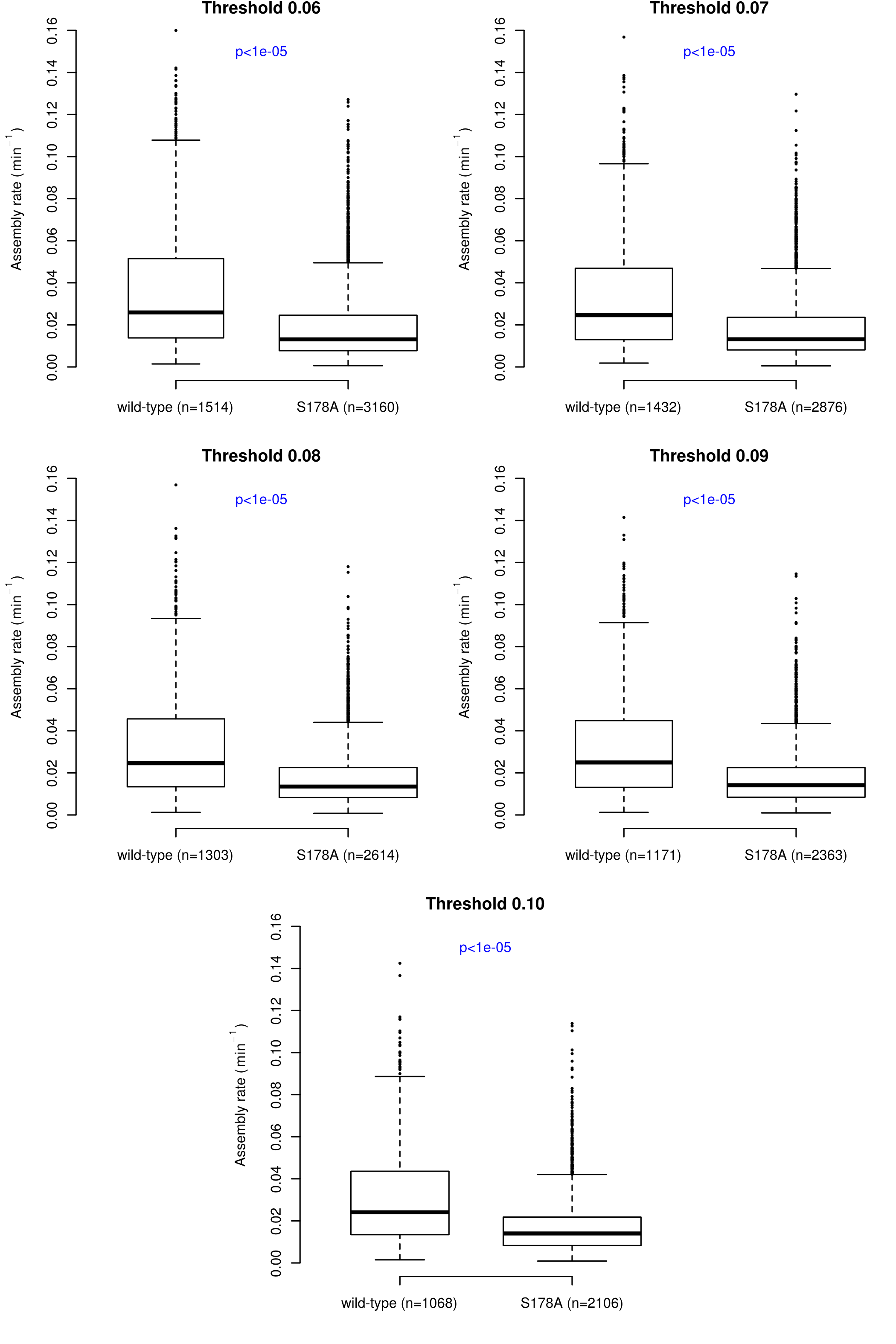

Supplement: Figure S5 — Changing the adhesion detection threshold does not affect the differences in the assembly rates between S178A mutant and wild-type cells. Each boxplot contains all the adhesions with significant linear fits (linear model p-value below 0.05). The p-values in each boxplot are for the difference in medians between the wild-type and S178A data sets in each boxplot. (PNG) [file pone.0022025.s005.png]

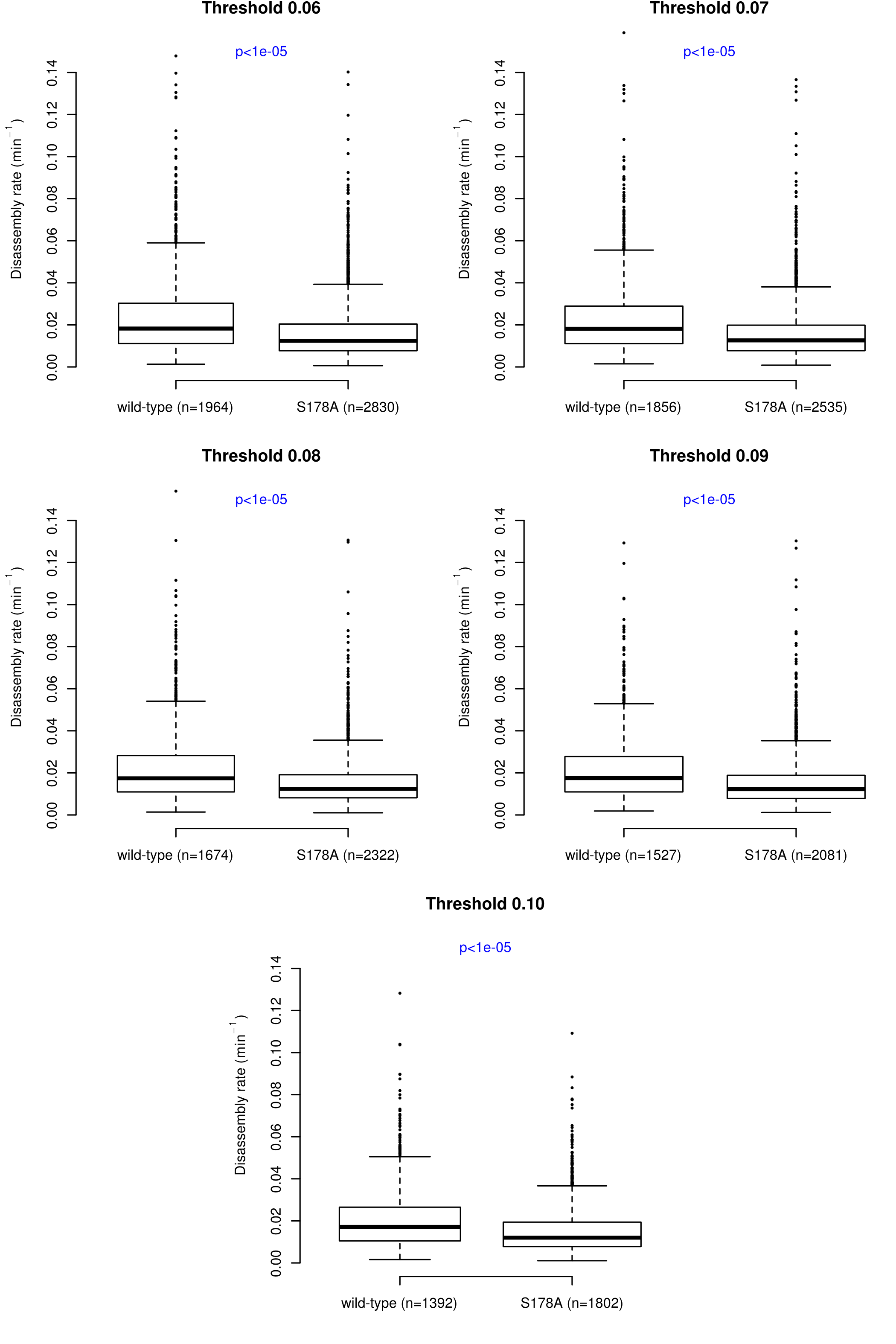

Supplement: Figure S6 — Changing the adhesion detection threshold does not affect the differences in the disassembly rates between S178A mutant and wild-type cells. Each boxplot contains all the adhesions with significant linear fits (linear model p-value below 0.05). The p-values in each boxplot are for the difference in medians between the wild-type and S178A data sets in each boxplot. (PNG) [file pone.0022025.s006.png]

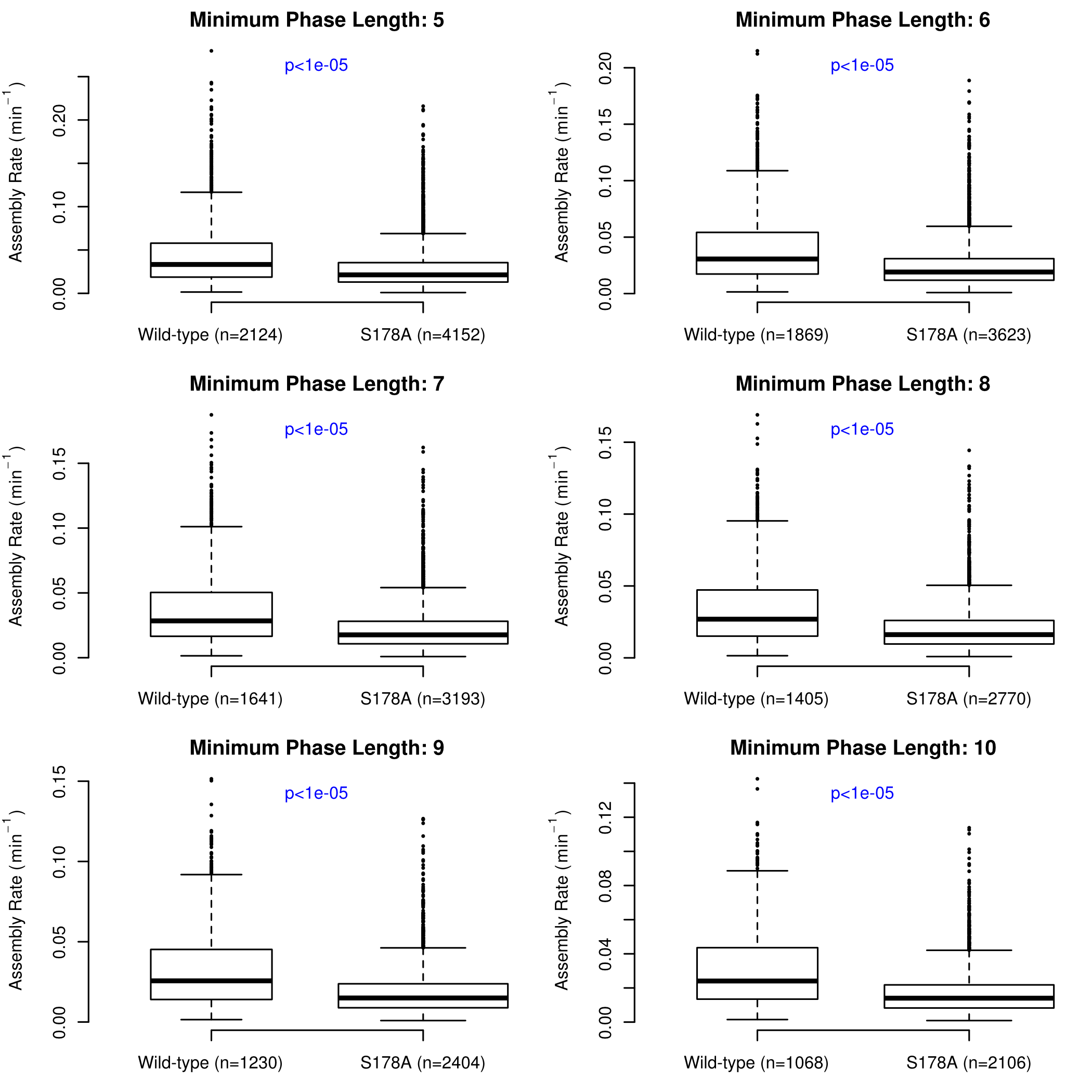

Supplement: Figure S7 — Changing the minimum length of the assembly phase does not significantly affect the differences in the assembly rate between the wild-type and S178A mutant cells. Each boxplot contains all the adhesions with significant linear fits (linear model p-value below 0.05). The p-values in each boxplot are for the difference in medians between the wild-type and S178A data sets in each boxplot. (TIFF) [file pone.0022025.s007.tif]

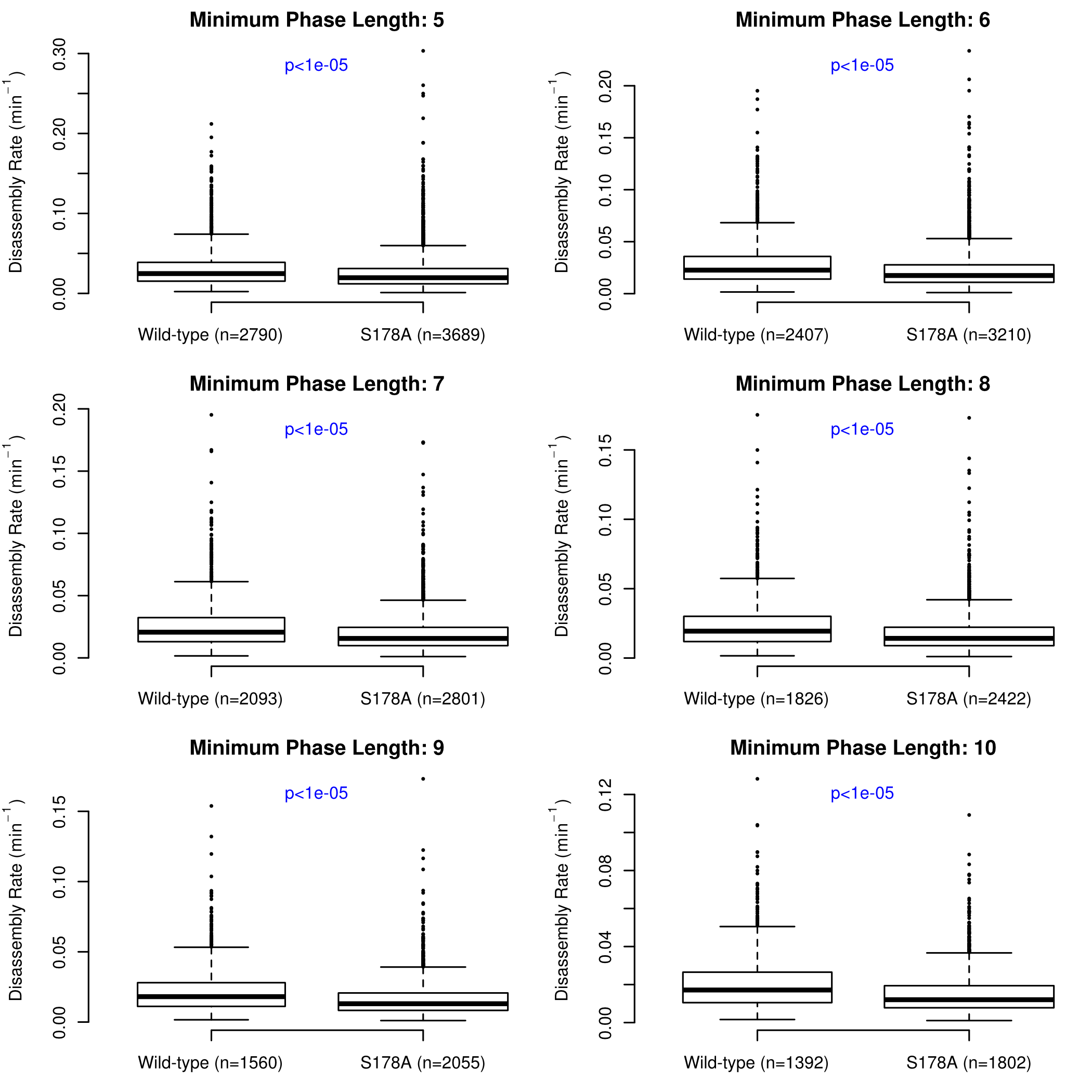

Supplement: Figure S8 — Changing the minimum length of the disassembly phase does not significantly affect the differences in the assembly rate between the wild-type and S178A mutant cells. Each boxplot contains all the adhesions with significant linear fits (linear model p-value below 0.05). The 95% confidence intervals on the percent change in the median assembly rate between the wild-type and S178A adhesions overlap in all minimum length settings. The p-values in each boxplot are for the difference in medians between the wild-type and S178A data sets in each boxplot. (PNG) [file pone.0022025.s008.png]

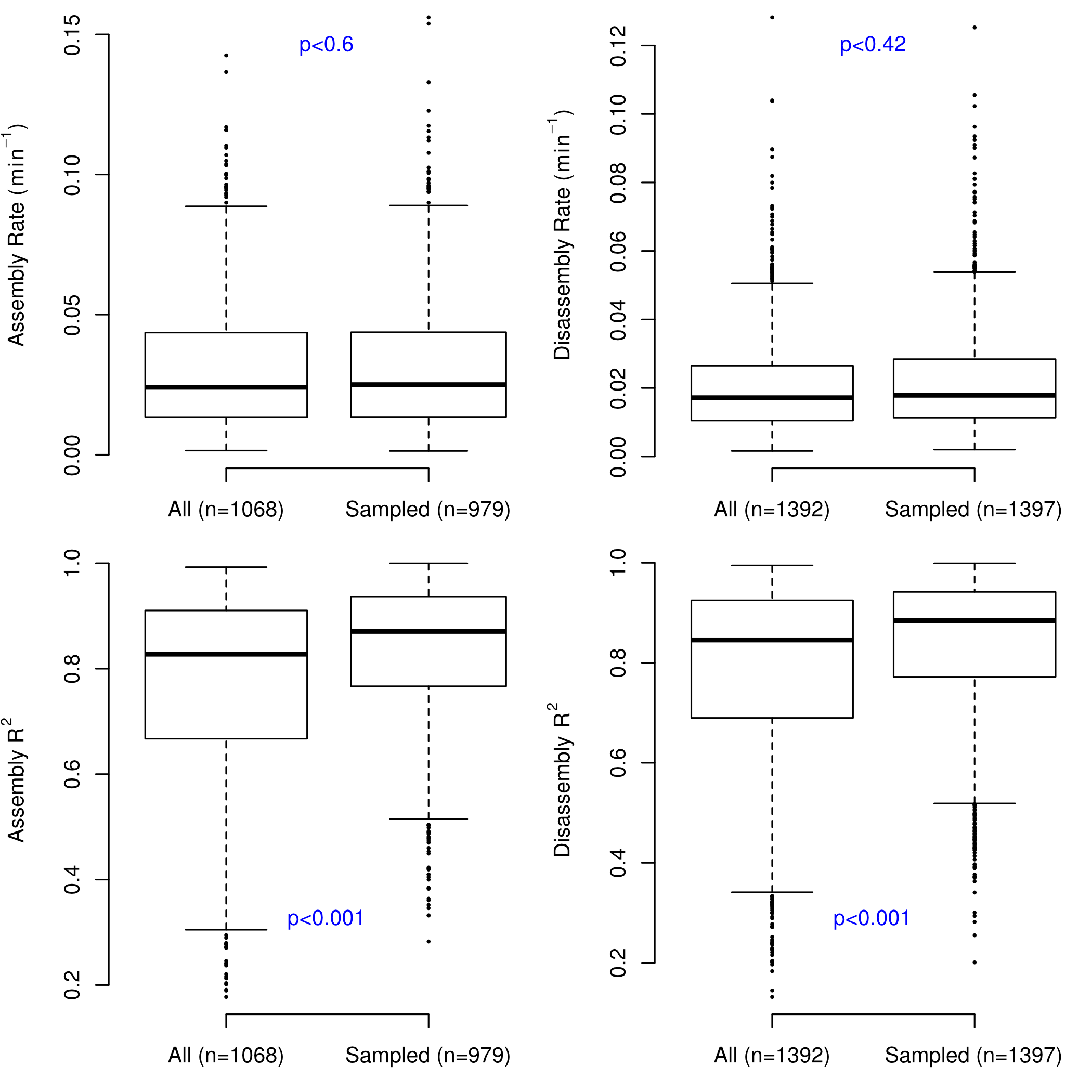

Supplement: Figure S9 — Reducing the time between each frame only has mild effects on the assembly and disassembly rates in the wild-type cells. The label ‘All’ indicates that none of the images were excluded to estimate the rates, while ‘Sampled’ indicates that every other image from each experiment was discarded. To compensate for the shortened experimental time, the minimum number of points needed to determine an assembly or disassembly rate was reduced to 5 for the sampled data sets. Each boxplot describes the data from all the adhesions with significant linear fits (p-value below 0.05). (PNG) [file pone.0022025.s009.png]

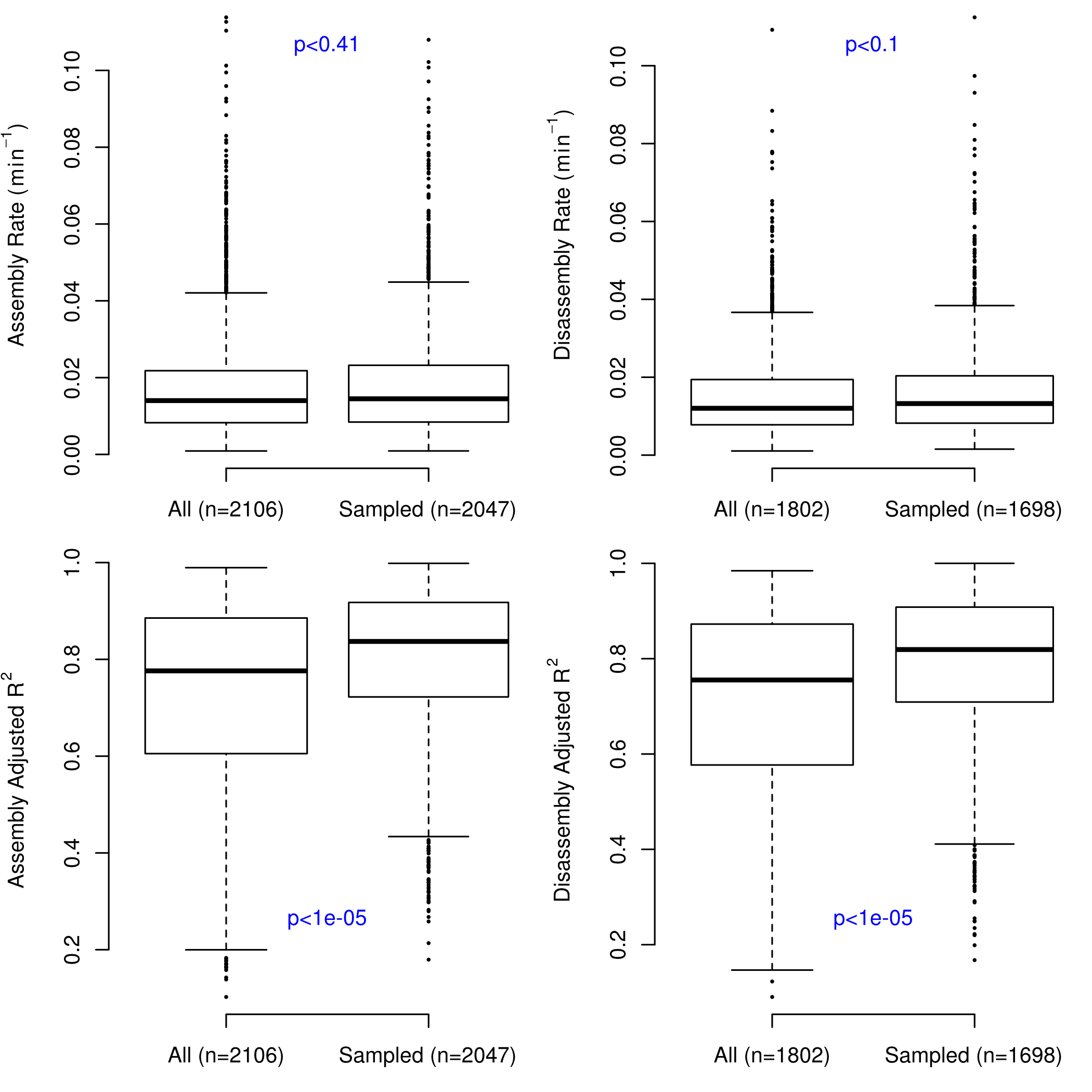

Supplement: Figure S10 — Reducing the time between each frame only has mild effects on the assembly and disassembly rates in the S178A cells. The label ‘All’ indicates that none of the images were excluded to estimate the rates, while ‘Sampled’ indicates that every other image from each experiment was discarded. To compensate for the shortened experimental time, the minimum number of points needed to determine an assembly or disassembly rate was reduced to 5 for the sampled data sets. Each boxplot describes the data from all the adhesions with significant linear fits (p-value below 0.05). (PNG) [file pone.0022025.s010.png]

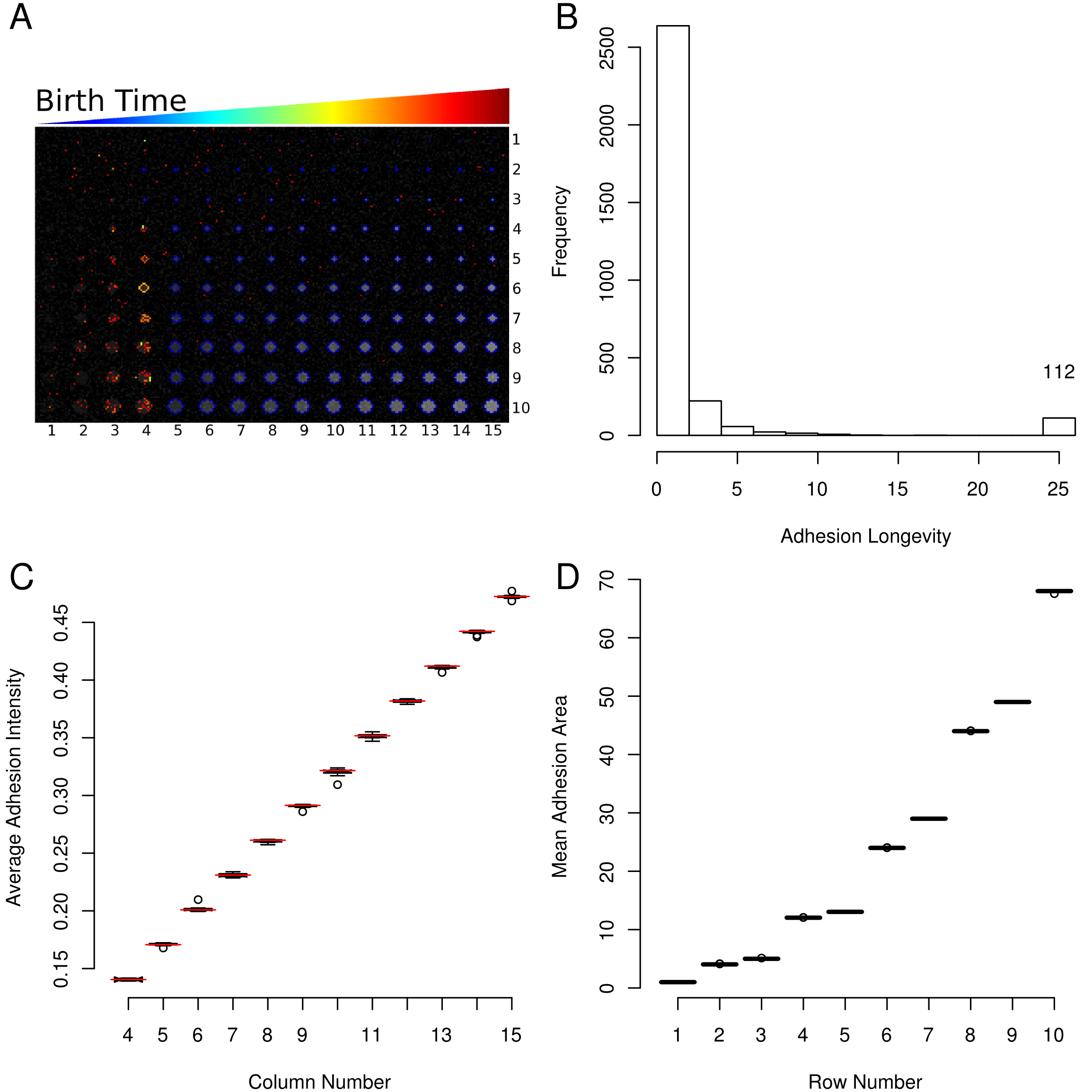

Supplement: Figure S11 — Evaluation of the analysis system's ability to extract quantitative properties from simulated stationary focal adhesions. (A) The last frame of the stationary simulation, with each adhesion outlined in a color depending on when in the movie it was born. The adhesions in blue have been detected for the longest time, while those in red and orange have been detected for the shortest amount of time. The simulated adhesions in columns 1–3 are all too faint to be reliably detected for the length of the simulation experiment, while those in column 4 are near the limit of detection. (B) The exponential distribution of adhesion longevity appears similar to that observed in the experimental data. The longevity of all the detected adhesions was correctly identified as 25 minutes. (C and D) The average adhesion intensity (C) and mean adhesion area (D) were correctly identified in the adhesions that were detected for their entire 25 minute lifespan. The red lines in C indicate the true values. (PNG) [file pone.0022025.s011.png]

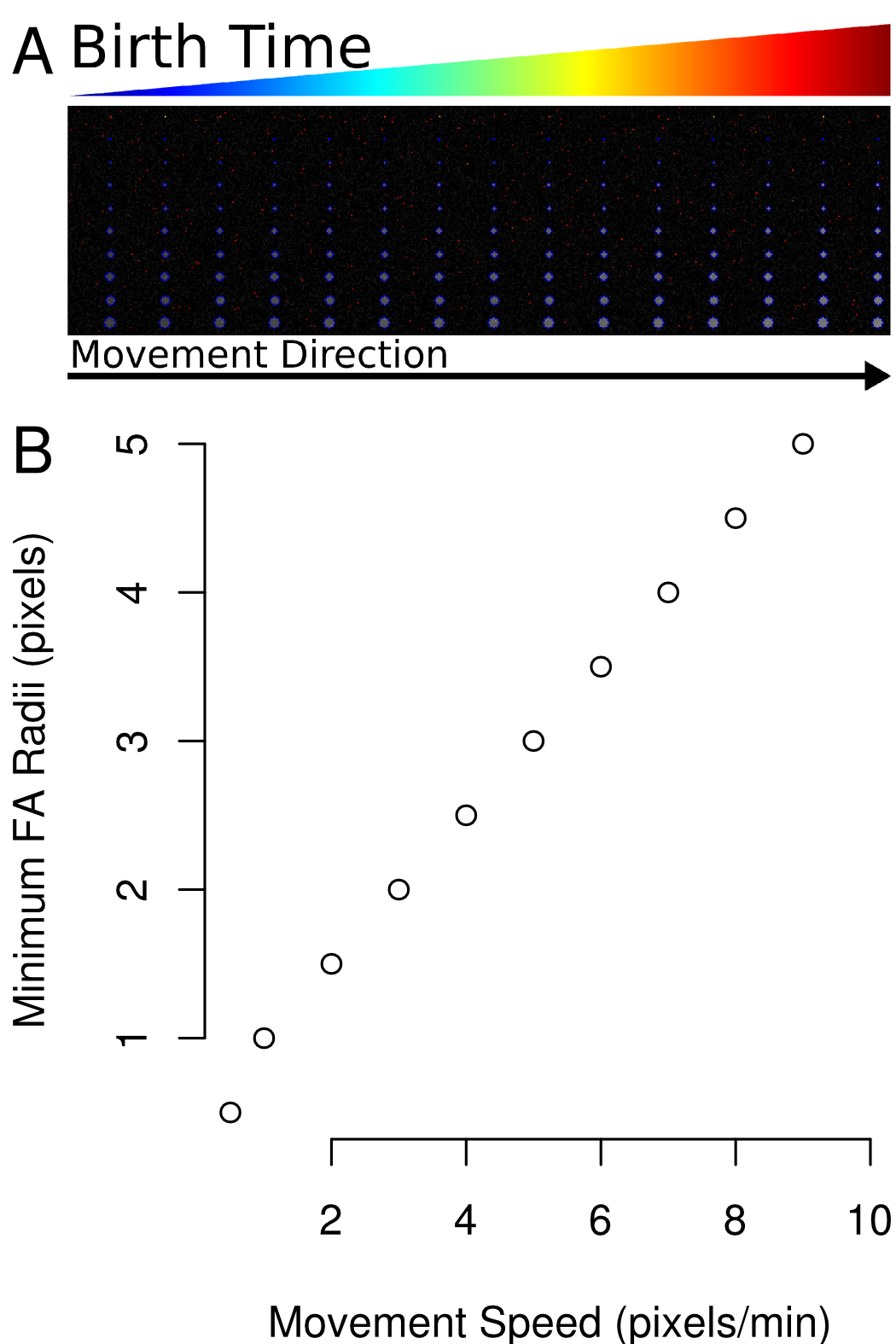

Supplement: Figure S12 — Evaluation of the tracking algorithm's ability to follow adhesions of various sizes and speeds. (A) A sample frame from the simulated adhesion motion experiment where the adhesions were moved at 1 pixel per frame. The top row of adhesions of only a single pixel could not be followed. (B) As the movement speed of the simulated adhesions increases, only larger adhesions can be reliably tracked. (PNG) [file pone.0022025.s012.png]

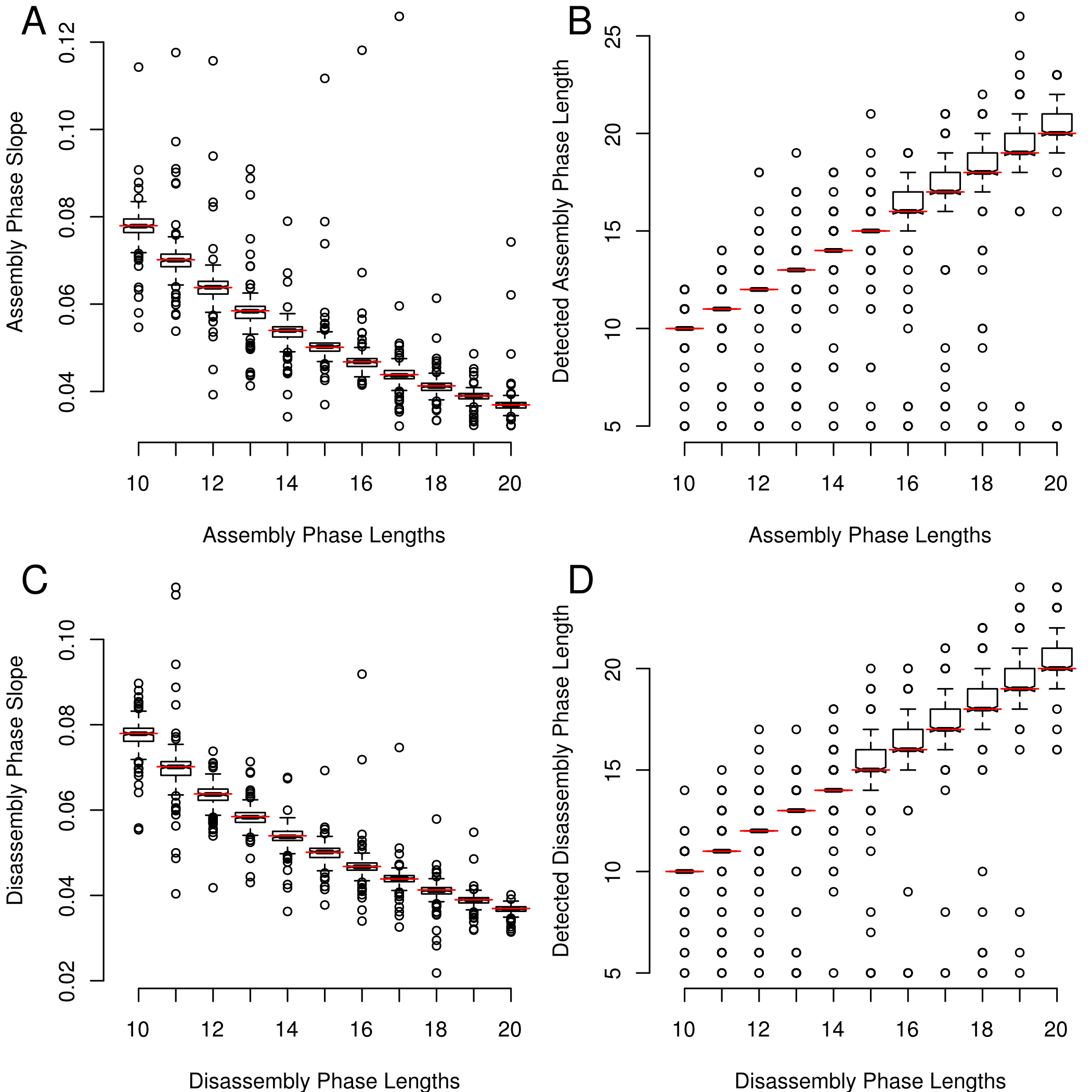

Supplement: Figure S13 — Evaluation of the rate and phase length detection algorithm using simulated focal adhesion images. (A and C) The predicted median assembly (A) and disassembly (C) rates were extracted correctly by the algorithm. (B and D) The predicted lengths of both the assembly (B) and disassembly (D) were also correctly identified by the algorithm. All the red lines indicate the expected values of the properties in each plot. (PNG) [file pone.0022025.s013.png]
